# Quantitative aortography for assessment of aortic regurgitation in the era of percutaneous aortic valve replacement

**DOI:** 10.3389/fcvm.2023.1161779

**Published:** 2023-07-17

**Authors:** Mahmoud Abdelshafy, Patrick W. Serruys, Tsung-Ying Tsai, Pruthvi Chenniganahosahalli Revaiah, Scot Garg, Jean-Paul Aben, Carl J. Schultz, Mohammad Abdelghani, Pim A. L. Tonino, Yosuke Miyazaki, Marcel C. M. Rutten, Martijn Cox, Cherif Sahyoun, Justin Teng, Hiroki Tateishi, Mohamed Abdel-Wahab, Nicolo Piazza, Michele Pighi, Rodrigo Modolo, Martijn van Mourik, Joanna Wykrzykowska, Robbert J. de Winter, Pedro A. Lemos, Fábio S. de Brito, Hideyuki Kawashima, Lars Søndergaard, Liesbeth Rosseel, Rutao Wang, Chao Gao, Ling Tao, Andreas Rück, Won-Keun Kim, Niels van Royen, Christian J. Terkelsen, Henrik Nissen, Matti Adam, Tanja K. Rudolph, Hendrik Wienemann, Ryo Torii, Franz Josef Neuman, Simon Schoechlin, Mao Chen, Ahmed Elkoumy, Hesham Elzomor, Ignacio J. Amat-Santos, Darren Mylotte, Osama Soliman, Yoshinobu Onuma

**Affiliations:** ^1^ Department of Cardiology, CORRIB Research Centre for Advanced Imaging and Core Laboratory, National University of Ireland, Galway (NUIG), Galway, Ireland; ^2^ Department of Cardiology, Al-Azhar University, Cairo, Egypt; ^3^ NHLI, Imperial College London, London, United Kingdom; ^4^ Department of Cardiology, Royal Blackburn Hospital, Blackburn, United Kingdom; ^5^ Pie Medical Imaging, Maastricht, Netherlands; ^6^ Department of Cardiology, Royal Perth Hospital, Perth, WA, Australia; ^7^ Medical School, University of Western Australia, Perth, WA, Australia; ^8^ Department of Cardiology, Amsterdam UMC, Amsterdam Cardiovascular Sciences, University of Amsterdam, Amsterdam, Netherlands; ^9^ Department of Cardiology, Catharina Hospital, Eindhoven, Netherlands; ^10^ Division of Cardiology, Department of Medicine and Clinical Science, Yamaguchi University Graduate School of Medicine, Yamaguchi, Japan; ^11^ Department of Biomedical Engineering, Eindhoven University of Technology, Eindhoven, Netherlands; ^12^ Xeltis B.V., Eindhoven, Netherlands; ^13^ Philips Healthcare, Best, Leipzig, Netherlands; ^14^ Department of Cardiology, Shibata Hospital, Yamaguchi, Japan; ^15^ Division of Cardiology, Department of Clinical Science and Medicine, Yamaguchi University Graduate School of Medicine, Ube, Japan; ^16^ Department of Internal Medicine/Cardiology, Heart Center Leipzig at the University of Leipzig, Leipzig, Germany; ^17^ Department of Medicine, Division of Cardiology, McGill University, Montreal, QC, Canada; ^18^ Division of Cardiology, Department of Medicine, University of Verona, Verona, Italy; ^19^ Boston Scientific, Maple Grove, MN, United States; ^20^ Department of Cardiology, University Medical Centre, Groningen, Netherlands; ^21^ Heart Institute (InCor), University of São Paulo Medical School (USP), São Paulo, Brazil; ^22^ Department of Cardiology, Teikyo University School of Medicine, Tokyo, Japan; ^23^ The Heart Centre, Rigshospitalet, Copenhagen University Hospital, Copenhagen, Denmark; ^24^ Department of Cardiology, Algemeen Stedelijk Ziekenhuis, Aalst, Belgium; ^25^ Department of Cardiology, Xijing Hospital, The Fourth Military Medical University, Xi'an, China; ^26^ Department of Cardiology, Karolinska Institute, Stockholm, Sweden; ^27^ Department of Cardiology, Kerckhoff Heart Centre, Bad Nauheim, Germany; ^28^ Department of Cardiology, Radboud University Medical Center, Nijmegen, Netherlands; ^29^ Department of Cardiology, Aarhus University Hospital, Aarhus, Denmark; ^30^ Department of Cardiology, Odense University Hospital, Odense, Denmark; ^31^ Department of Cardiology, Faculty of Medicine, Heart Center, University of Cologne, Cologne, Germany; ^32^ Department for General and Interventional Cardiology/Angiology, Heart- und Diabetes Center NRW, Ruhr-Universität Bochum, Bad Oeynhausen, Germany; ^33^ Department of Mechanical Engineering, University College London, London, United Kingdom; ^34^ Division of Cardiology and Angiology II, University Heart Centre Freiburg—Bad Krozingen, Bad Krozingen, Germany; ^35^ Department of Cardiology, West China Hospital, Sichuan University, Chengdu, China; ^36^ Islamic Center of Cardiology and Cardiac Surgery, Al-Azhar University, Cairo, Egypt; ^37^ CIBERCV, Cardiology Department, Hospital Clínico de Valladolid, Valladolid, Spain

**Keywords:** aortic regurgitation, paravalvular leak, videodensitometry, transcatheter aortic valve replacement, transcatheter aortic valve implantation, quantitative aortography

## Abstract

Paravalvular leak (PVL) is a shortcoming that can erode the clinical benefits of transcatheter valve replacement (TAVR) and therefore a readily applicable method (aortography) to quantitate PVL objectively and accurately in the interventional suite is appealing to all operators. The ratio between the areas of the time-density curves in the aorta and left ventricular outflow tract (LVOT-AR) defines the regurgitation fraction (RF). This technique has been validated in a mock circulation; a single injection in diastole was further tested in porcine and ovine models. In the clinical setting, LVOT-AR was compared with trans-thoracic and trans-oesophageal echocardiography and cardiac magnetic resonance imaging. LVOT-AR > 17% discriminates mild from moderate aortic regurgitation on echocardiography and confers a poor prognosis in multiple registries, and justifies balloon post-dilatation. The LVOT-AR differentiates the individual performances of many old and novel devices and is being used in ongoing randomized trials and registries.

## Highlights

• Aortic regurgitation following TAVR negatively affects patient outcomes.
• Video-densitometry is an objective, accurate, and well-validated tool for aortic regurgitation adjudication.
• LVOT-AR > 17% has a poor prognosis and justified further intervention.
• Many TAVR devices have been evaluated using video-densitometry, and the technique is currently being used in ongoing trials and registries.

## Quantitative aortography for regurgitation assessment after TAVR: an unmet need

The clinical indications, vascular approaches, and technologies of transcatheter aortic valve replacement (TAVR) are continuously evolving, and as its use increases exponentially, the abrogation of major procedural shortcomings becomes imperative. Among the imperfect results, paravalvular leak (PVL) is one that contributes considerably to the erosion of clinical benefits with its adverse effects on mortality, morbidity, and reverse cardiac remodeling. Consequently, a simple method, readily applicable in the interventional suite to timely detect and quantitate PVL objectively, reproducibly, and accurately remains the wish and goal of the “minimalist” operator.

Trans-thoracic echocardiography (TTE) is a safe and convenient tool that provides real-time information on the severity of trans- or para-valvular aortic regurgitation (AR) and is thus the preferred method of assessing and monitoring PVL. However, TTE is not exempt from methodological drawbacks such as the significant influence of the imaging plane and a lack of consistency in the severity of PVL described between core labs and between imaging modalities (Supplementary Figure S1) (1).

In the era of minimalist TAVR the use of angiography has become more common as it provides PVL grading as the sum of all jets regardless of their number, level, or trajectory, furthermore as it is performed immediately post implant, any significant PVL can be corrected by device post-dilatation, repositioning, or implantation of a second valve. Interventional cardiologists are familiar with this technique, which is fast, readily available, and part of their technical environment, however, angiographic grading is qualitative and subjective. These issues highlighted the need for a quantitative assessment of PVL from aortography. In this comprehensive narrative review, we delve into the principles and history of video-densitometric assessment of aortic regurgitation, discussing its *in vitro* and *in vivo* validation, correlation with other imaging modalities, prognostic value, current limitations, and potential for future development. To find relevant articles for this narrative review, we searched MEDLINE and Embase for articles in English language with the following terms: “Paravalvular leak”, “PVL”, “aortic regurgitation”, “AR”, “Transcatheter aortic valve implantation”, “TAVI”, “transcatheter aortic valve replacement”, “TAVR”, “video-densitometry”, “aortography”, and “Quantitative Aortography”.

## Principle and history of video-densitometry

In the eighties, long before the TAVR era, aortography was dethroned by echocardiography for the iterative and non-invasive assessment of AR, however, aortography was actually already “quantitative” at the time, and several attempts had been made to make the assessment of AR by aortography more objective, less categorical and more numerically quantitative (2–5). At that time, video-densitometric analysis was used for the evaluation of native AR; moreover, it had also been used for the assessment of AR after balloon aortic valvuloplasty (Supplementary Figure S2) (2).

## The concept of video-densitometry and phantom validation

**Figure 1:**
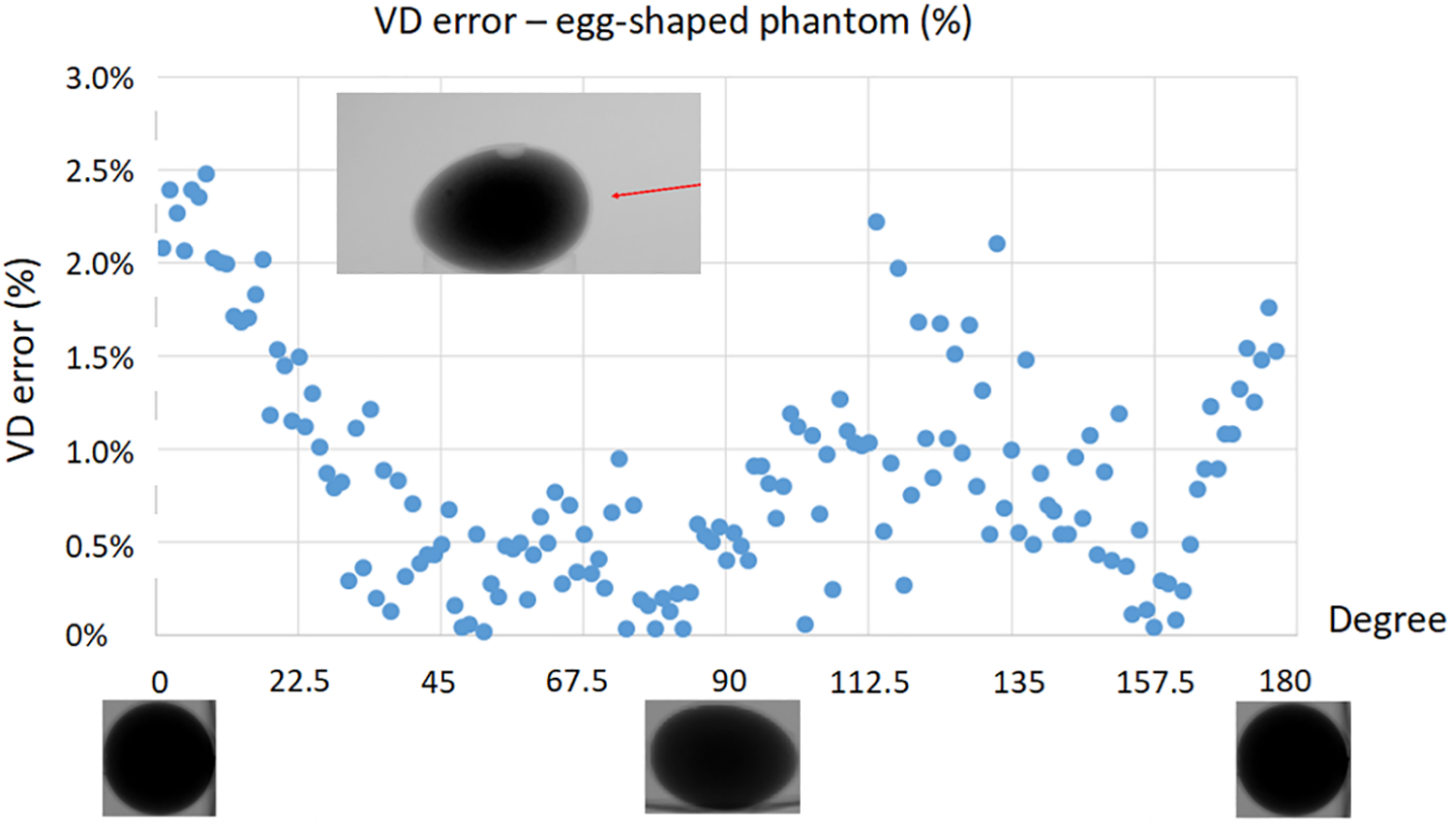
Video-densitometric assessment of an egg-shaped plastic phantom filled with radiopaque contrast medium rotated in an x-ray field from short (0, 180 degree) to long axis view (90 degree). [Modified From Kawashima, et al. (6)].

The most common objection raised by clinicians regarding the methodology of video-densitometry is the potential for measurement errors due to an assumed parallax effect when the outflow tract is assessed from various angiographic views. However, the approach is aimed at circumventing this issue; a video-densitometric measurement is the constant product of an area in which the radiopaque contrast is detected, multiplied by the density (hemodilution) of the radiopaque contrast medium in that area/volume (6). This means that the x-ray attenuation of an object is virtually unaffected by the viewing direction (angulation) of the x-ray system. As illustrated in Figure 1, the error in the video-densitometric measurement, as demonstrated using an egg-shaped plastic phantom filled with angiographic medium and rotated in the radiation field, is <2.5% (maximal video-densitometric error) even at extreme parallax angles. Notably, this does not compromise the accuracy in assessing regurgitation values in clinical practice, which range from >6% for mild regurgitation to >17% for moderate/severe regurgitation.

In 2014, Schultz et al. (7) used video-densitometry and pioneered the concept of quantitative Seller's assessment for regurgitation (qRA index) by quantifying the density, duration, and depth of LV opacification. Ultimately this yields a continuous severity scale of regurgitation ranging from grade 0, which indicates no contrast leakage into the LV, to grade 4 that indicates contrast filling the entire left ventricle (LV), with the density of angiographic contrast medium higher in the LV than the aortic root.

However, the criteria for analyzability were only met in 15% (*n* = 44) of 285 retrospective aortograms and in 69% (*n* = 22) of 32 prospectively collected aortograms. Nevertheless, the visual grade of AR (Sellers) was highly correlated with the time-density measurements including the Relative Area Under the Curve (RAUC) and qRA index (*r* = 0.81 and 0.83, respectively, *p* < 0.001). Inter-observer reproducibility of visual grading was moderate (kappa 0.47–0.60, *p* < 0.001). Inter-observer measurement of RAUC and qRA index were highly correlated (*r* = 0.98, *p* < 0.001) and showed a high level of agreement (0.00 ± 0.25).

In 2016 Tateishi et al. (8) proposed changing the region of interest (ROI) as interrogation of the entire LV was not always feasible. Indeed, the apex was sometimes not visualized, or there were overlapping structures in LV ROI which influenced the video-densitometric analysis such as a contrast in the descending aorta, radiopaque objects such as the trans-esophageal echocardiography (TEE) probe, a high position of the diaphragm, and gastric or bowel gas etc. To avoid the interference of such radiopaque structures, the left ventricle outflow tract (LVOT) was selected as an alternative ROI.

Briefly, the software (CAAS A-valve, Pie Medical Imaging, Maastricht, the Netherlands) constructs two time-density curves assessed in two ROI: the aortic root where contrast is injected and the LVOT where the regurgitation is quantified. To define the LVOT area a line is drawn from the valve plane to the apex, subdivided in three equal regions; the upper region is the ROI for the video-densitometric assessment of regurgitation in the LVOT. The ratio between the areas under the two-time density curves of these regions over at least three cardiac cycles is the Regurgitation Fraction expressed in percentage.

**Figure 2:**
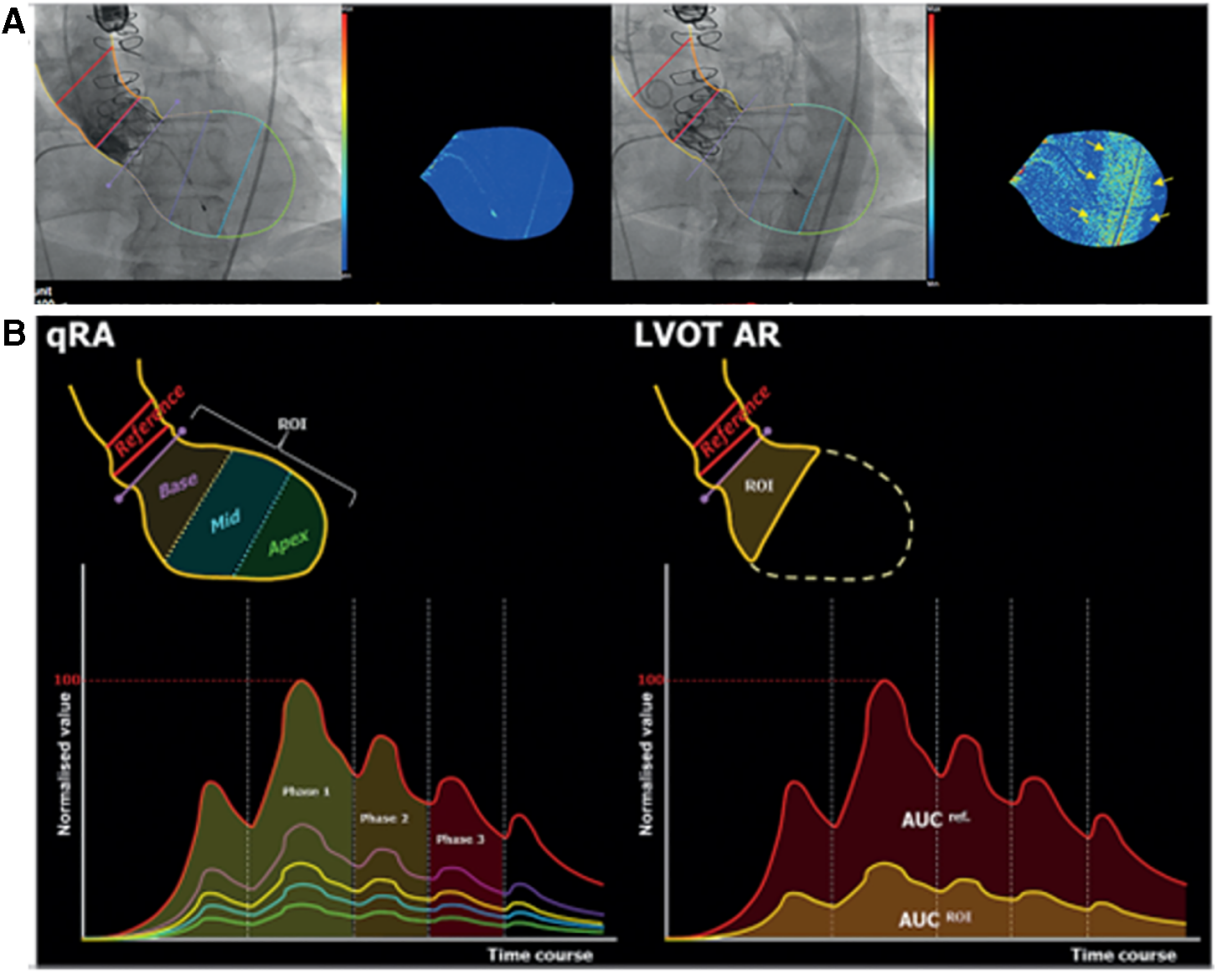
(**A**) Trace of aortic regurgitation (upper left panel) just after contrast injection. Later, the contrast-filled descending aorta overlaps the LV (upper right panel), causing a spurious increase in contrast density and colour-density map. (**B)** time-density curves (TDC) of qRA index (left)and LVOT-AR. (right panel). [see text, modified from Tateishi, et al. (8)].

This new approach was called “LVOT-AR”—left ventricular outflow tract-aortic regurgitation (Figure 2) –and its feasibility and reproducibility was investigated in 182 aortograms taken as part of the Brazilian TAVR registry. LVOT-AR was analyzable in 64.8% of aortograms vs. 29.7%, when using the entire LV gram and qRA index.

Using the LVOT-AR, inter-observer variability in a Bland Altman analysis was low [mean difference ± standard deviation (SD): 1 ± 5% *p* = 0.53], and the two observers’ measurements highly correlated (Pearson’s coefficient of correlation, *r* = 0.95, *p* < 0.001).

## Video-densitometry validation of LVOT-AR

### *In vitro* and *vivo* validation

Quantitative assessment of PVL by video-densitometry of aortograms correlates strongly with the actual regurgitation fraction (RF) as measured by a flow probe (Transonic 28PAU, with TS410 flowmeter; Transonic, Ithaca, NY, USA) in a controlled *in vitro* mock circulation model (9) and in a porcine model (10) setting as described in the Supplementary Data Sheet and illustrated in Supplementary Figures S3, S4.

### Using of synchronized injection to minimize the amount of contrast

One of the main limitations of aortography in TAVR patients -who commonly have some degree of kidney dysfunction- is the large amount of contrast medium injected during a conventional fluoroscopic acquisition, which may subsequently contribute to the risk of periprocedural acute kidney injury (AKI) portending a poor outcome after TAVR (11).

In clinical practice, aortography is performed with a contrast injection (15–25 ml) which typically lasts for 1–2 s, covering a few cardiac cycles. Given the fact that PVL is a diastolic event, a short injection during only diastole could avoid the wash-out of contrast by the stroke volume ejected into the aorta during systole (when the aortic valve is open) and, consequently, reduce the contrast volume required per acquisition down to 8 ml (12). This concept was tested *in vitro* by Miyazaki et al. (12) with an ACIST CVi® contrast delivery system (ACIST Medical Systems, Eden Prairie, MN, USA), and then in a porcine model by Modolo et al. (10) which confirmed the feasibility of synchronized diastolic injection. The detailed methodology of the synchronized injection validation is described in the Supplementary Data Sheet and in Supplementary Figures S5, S6. However, it is unfortunate that manufacturers involved with the technology of pump injectors have not yet fully appreciated this unmet need in TAVR procedures and the clinical value of a greatly reduced contrast volume administered during a diastolic aortogram triggered by the QRS complex and strictly synchronized to a single period of diastole, thus, currently, no human study of this synchronized injection has been performed.

## Correlation of LVOT AR with clinical trans thoracic echocardiographic assessment post TAVR

**Figure 3:**
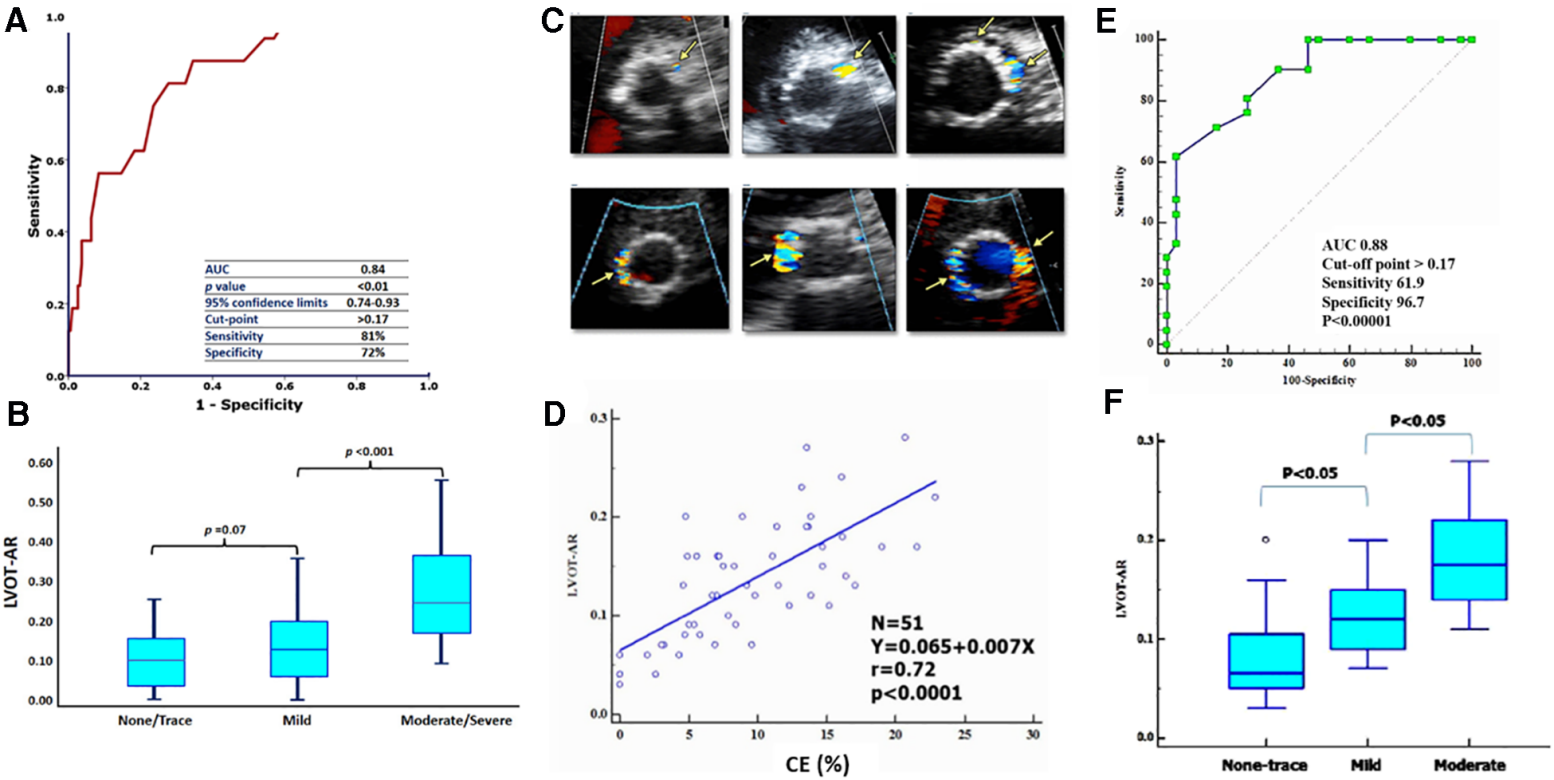
ROC curve of LVOT-AR corresponding to greater than mild post-TAVR AR on intra-procedural TTE (**A**) and TEE (**E**). LVOT-AR values (median and quartiles) post-TAVR correlated to four categories of regurgitation on TTE (**B**) and TTE (**F**). (**C,D**) circumferential extent (CE %) of different degrees of AR on TEE and Linear regression of LVOT-AR with CE % [modified from Tateishi, et al. (15) and Abdelghani, et al. (14)].

In 2017, LVOT-AR was quantitated in 228 consecutive patients enrolled in the TAVR Brazilian registry before, and after, TAVR by echocardiography, and by video-densitometric analysis of aortograms after TAVR (13). Post TAVR LVOT-ARs of 10.6 ± 8%, 13.6 ± 10% and 28.6 ± 14% were respectively measured in none-trace, mild and moderate-severe post-TAVR AR as defined by echocardiography (*p* < 0.001). An LVOT-AR of 17%—corresponding to the Youden index on the area under the curve (AUC = 0.84, sensitivity 81% and specificity 72%), is to the best cut off criteria discriminating trace-mild AR from moderate-severe AR as diagnosed on TTE Figures 3A,B.

In the more recent RESPOND registry (NCT02031302) using the Lotus valve analysis of 472 consecutive aortograms confirmed the excellent correlation between TTE and LVOT-AR (14). There were significant differences in LVOT-AR across the different strata of echo-PVL: 2.0% [0.0% to 4.0%] vs. 3.0% [1.0% to 7.0%] vs. 3.0% [1.75% to 9.25%] vs. 7.0% for none, trace, mild, and moderate PVL, respectively (*p* < 0.001)**.**

Based on previous validation, the following cut of points were used: LVOT-AR > 17% for moderate/severe AR, 6%≤ LVOT-AR ≤17% for mild AR and LVOT-AR < 6% representing the normal closing volume of the aortic valve.

## Correlation of LVOT-AR with periprocedural TEE and TTE pre-discharge

In 2018, Tateishi et al. performed blinded retrospective comparisons of intra-procedural video-densitometry with intra-procedural TEE and pre-discharge TTE (15). Fifty-one periprocedural TEE and LVOT-AR by video-densitometry were both evaluable. The circumferential extent (CE) of the paravalvular regurgitant jet was measured on echocardiography and graded according to the VARC-2 criteria. The overall median LVOT-AR on video-densitometry was 13% (IQR 9%–17%) with the median LVOT-AR 7% (IQR 5%–11%), 12% (IQR 9%–15%), and 17% (IQR 15%–22%) in no-trace (*n* = 12), mild (*n* = 18), and moderate (*n* = 21) PVL, respectively, as defined by the %CE on intra-procedural TEE, while it was 8% (IQR 7%–12%), 13% (IQR 12%–16%), and 17% (IQR 12%–20%) in no-trace (*n* = 13), mild (*n* = 14), and moderate (*n* = 20) PVL, respectively, as defined by %CE on pre-discharge TTE. There was a strong correlation between LVOT-AR and %CE on intra-procedural TEE (*r* = 0.72, *p* < 0.0001), while there was a moderate correlation between LVOT-AR and %CE on pre-discharge TTE (*r* = 0.49, *p* = 0.0005) Figures 3C–F.

## Correlation of LVOT AR with cardiac magnetic resonance regurgitation fraction (CMR-RF)

In 2018, Abdel-Wahab et al. compared LVOT-AR by video-densitometry to CMR-derived regurgitation fraction (CMR-RF) for the quantification of PVL in 135 patients after TAVR (16). The average CMR-RF was 6.7 ± 7.0% whereas the average LVOT-AR was 7.0 ± 7.0%; with a substantial correlation (*r* = 0.78, *p* < 0.001). On receiver-operating characteristic curves, an LVOT-AR ≥10% corresponded to > mild PVL as defined by CMR-RF (AUC: 0.94; *p* < 0.001; sensitivity 100%, specificity 83%), whereas an LVOT-AR ≥25% corresponded to moderate-to-severe PVL (AUC: 0.99; *p* < 0.004; sensitivity 100%, specificity 98%). Intra-observer reproducibility was excellent for both techniques (CMR-RF, intraclass correlation coefficient: 0.91, *p* < 0.001; LVOT-AR intraclass correlation coefficient: 0.93, *p* < 0.001). These results confirm that LVOT-AR provides a surrogate assessment of PVL severity after TAVR that correlates well with the CMR-RF Supplementary Figure S7.

## Regurgitation fraction of 17% as the threshold criteria for post dilatation

**Figure 4:**
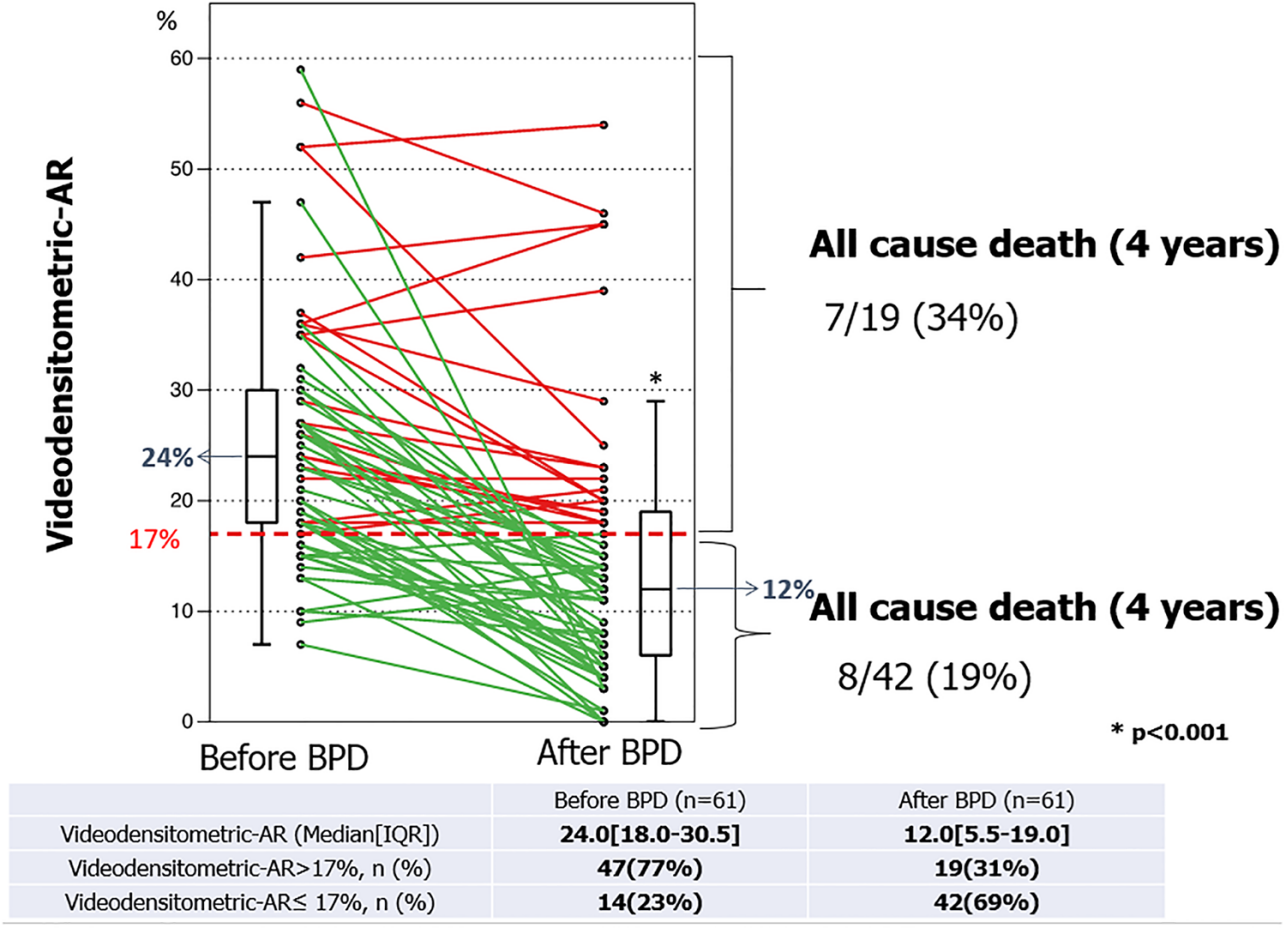
Individual LVOT-AR serial changes before and after balloon post-dilatation. At 4 years, in patients with LVOT-AR > 17%, 7 deaths (34%) occurred, whereas in patients with VD-AR ≤ 17%, 8 deaths (19%) were observed [modified from Miyazaki et al. (17)].

In 2018 the Brazilian TAVR registry reported a significant reduction in LVOT-AR from 24.0 (18.0–30.5) % to 12.0 (5.5–19.0) % after balloon post-dilatation (BPD) in 61 patients with some degree of PVL (*p* < 0.001), among their cohort of 399 patients who had undergone TAVR. The relative delta of LVOT-AR after BPD ranged from −100% (improvement) to +40% (deterioration) and its median value was −46.2%. The frequency of improvement, no change, and deterioration were 70% (*n* = 43), 25% (*n* = 15) and 5% (*n* = 3), respectively. Significant AR (LVOT-AR > 17%) was observed in 47 patients (77%) before and in 19 patients (31%) after BPD. These results confirmed that LVOT-AR after TAVR provides a quantitative assessment of post-TAVR regurgitation and can help in deciding whether to perform a BPD and determine its efficacy Figure 4. Indeed, online video-densitometric assessment of AR in the cath lab has been proven feasible in over 92% of cases through multiple studies described in the following section (17, 18). However, whether BPD, according to intra-procedural assessments of LVOT-AR > 17% will provide a better outcome for patients remains to be proven with prospective clinical trials. Indeed, the OVAL GUIDE trials, as described in detail in the following sections, are designed to fill this evidence gap.

## Regurgitation fraction of 17% as a vital prognostic determinant

In 2016 Tateishi et al. (8) established the prognostic impact of an LVOT-AR > 17% when he reported that these patients had a significantly higher one-year all-cause mortality compared with those with LVOT-AR ≤17% (37.1% vs. 11.2%, *p* = 0.0008, Central Illustration A). The poor prognosis in this group was subsequently confirmed in the Brazilian TAVR registry by Abdelghani et al. (13) who reported a significantly higher mortality at 30-days (16.4% vs. 7.1%, *p* < 0.035) and 1-year [32.9% vs. 14.2%, log rank *p* < 0.001; HR: 2.690 (1.461–4.953), *p* < 0.001] among patients with an LVOT-AR > 17% compared to an LVOT-AR ≤ 17% (Central Illustration B) (13).

The rate of the composite endpoint of all-cause death or re-hospitalization for heart failure at 1-year was significantly higher in patients with moderate/severe AR compared with trace/mild AR on intra-procedural echocardiography (41.5% vs. 12.4%, *P* = 0.03) as well as in patients with an LVOT AR > 17% vs. an LVOT-AR ≤ 17% (59.5% vs. 16.6%, *P* = 0.03) (15) Central Illustration C.

This prognostic value of LVOT-AR > 17% was confirmed at 1296 days follow up [68.3% vs. 39.5%, log rank *p* = 0.0023, HR 2.64 (1.27–5.50), *p* = 0.0023] (8) Central Illustration A.

LV remodelling: At a median of 496 days follow-up patients with an LVOT-AR ≤ 17% had a significant reduction in LV mass index (LVMi; from 140 [112– 169] to 121 [95–148] g/m^2^, *p* < 0.009) and the prevalence of LV hypertrophy (LVH; from 88 to 64%, *p* < 0.001) compared to baseline. In contrast, among patients with an LVOT-AR > 17%, the LVMi (from 149 [121–178] to 166 [144–188] g/m^2^, *p* = 0.14) and the prevalence of LVH (from74 to 87%, *p* = 0.23) evolved in the opposite direction and failed to show any significant change (13).

Regarding the long-term prognostic impact of mild PVL,-assessed by video-densitometry (LVOT-AR)-, on the prediction of 5-year mortality, a preliminary analysis shows diverging Kaplan-Meyer curves and suggest a potential mortality discrimination between none/trace (LVOT-AR 0%-2%), mild (LVOT-AR 3%-16%) and moderate/severe (LVOT-AR >16%)regurgitation (statistically not significant *p* = 0.132) Central Illustration D**.** Of note the cut-off criteria of video-densitometric regurgitation vs. categorical echocardiographic assessment of none/trace, mild, and moderate/severe regurgitation has been specifically determined in a blind fashion (CORRIB lab) by C statistic and AUC in a cohort of patients studied at Bad Krozingen (Courtesy of Prof Neuman and dr Schoechlin) (19).

## Feasibility of analysis, protocol of acquisition and yield of data

LVOT-AR by video-densitometry has been evaluated in comparison with TTE, TEE and CMR using a variety of THV devices. However, due to the retrospective nature of these analyses and the absence of any specific acquisition protocols, analyses were only feasible in 57.1% and 57.5% of the patients enrolled in the respective Brazilian TAVR Registry (8, 20) and the RESPOND study (14), leaving a large proportion of patients unaccounted for.

The major issue impacting on the feasibility of retrospective LVOT-AR assessment is the overlap of the descending aorta with either the ROI (LVOT) or the reference area (aortic root). The software analyses the density changes over time in both regions, thus any other change in density in the background of the RX acquisition due to contrast passing through the descending aorta and overlapping with these regions impacts on the final result. Although the analyses are still technically possible in these situations, the results would not reflect the true regurgitation; therefore, they are considered non-analysable.

**Figure 5:**
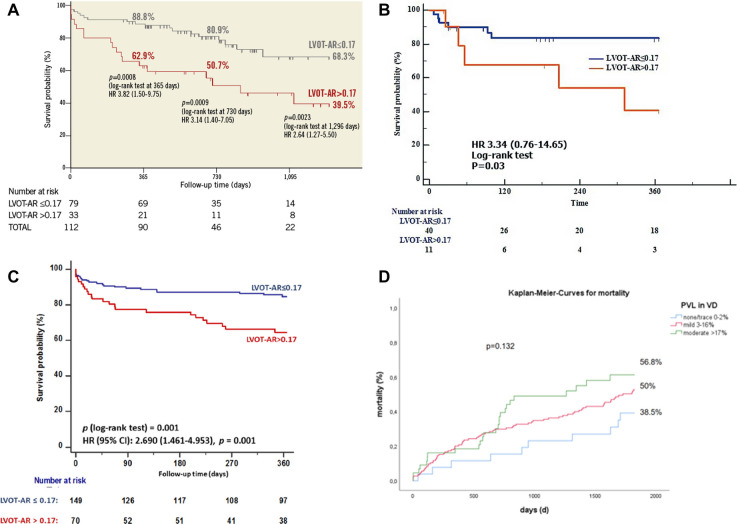
Central illustration. Kaplan-Meier estimates of cumulative survival after TAVR at 30 days, 1 year four years and five years stratified according to the degree of LVOT-AR. Compiled illustration (**A**,**B**) from Tateishi et al. (8, 15) (**C**) from Abdelghani, et al. (14) and (**D**) from Bad Krozingen TAVI cohort [partially unpublished data (16)].

**Figure 6:**
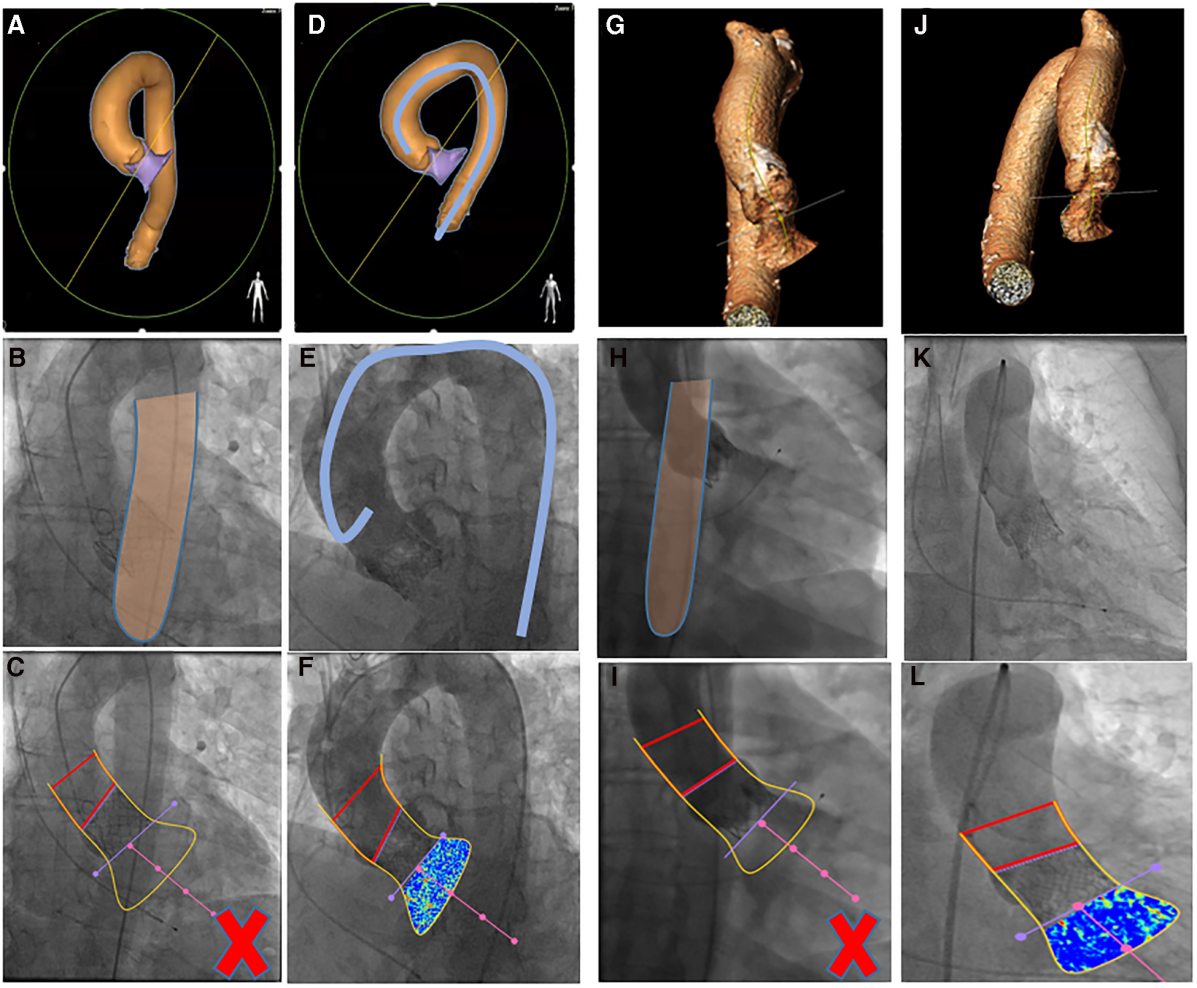
Overlap of either outflow tract (**A**) or ascending aorta (**G**) on descending aorta visualized by MSCT [heart navigator software (**A**) or 3mensio software (**G**)] and by aortography, invalidating the video-densitometric assessment (**C,I**). Further rotation either toward LAO (**D**) or RAO (**J**) resolves the overlapping issue (**F,L**). **(D,E,K**) use of intravascular catheter to avoid overlapping [Teng's rule (22), see text].

Methods to improve the feasibility of video-densitometry include using pre-procedural multi-slice computed tomography (MSCT) to optimize the angiographic projection, and this was first tested at the Yamaguchi University along with other directives to optimise the angiographic acquisition protocol for video-densitometry Table 1 and Figures 5, 6.

**Table 1:** Summary of angiographic acquisition protocol for video-densitometry.

Image acquisition environment
With ECG recording
Image acquisition should take ≥2 beats before contrast injection for subtracting the static background
Image acquisition should take ≥3–5 heartbeats after contrast injection
Cooperation with patients and other staff
No table or patient motion during image acquisition
Breathing hold during image acquisition
Angiographic projection
Avoid contrast-filled descending aorta overlap with the LVOT (ROI)
Avoid contrast-filled descending aorta overlap with the aortic root (reference)
Be sure radio-dense objects do not over-project on ROI
Avoid the diaphragmatic over-projection
Contrast and catheter
5-Fr pigtail catheter located ±20 mm from the inflection point of Self Expandable Valves (SEV) and top of Balloon Expandable Valve (BEV) but not interfering with the prosthesis leaflets
Contrast injection speed 10 ml/sec, volume 20 ml

In 92 consecutive patients from the Yamagishi University, post-TAVR LVOT-AR was assessed in two sequential cohorts investigated either prior to (*n* = 54) or following (*n* = 38) implementation of a standardized acquisition protocol. The protocol involved using MSCT for planning the optimal angiographic projection, and its use led to the feasibility of video-densitometry improving from 57.4% to 100% Figure 6. In 69 analysable aortograms (69/92; 75%), LVOT-AR ranged from 3% to 28% (median 12%). Inter-observer agreement was high (mean difference ± SD, 1 ± 2%) with the two observers’ measurements highly correlated (*r* = 0.94, *p* < 0.0001) (21).

**Figure 7:**
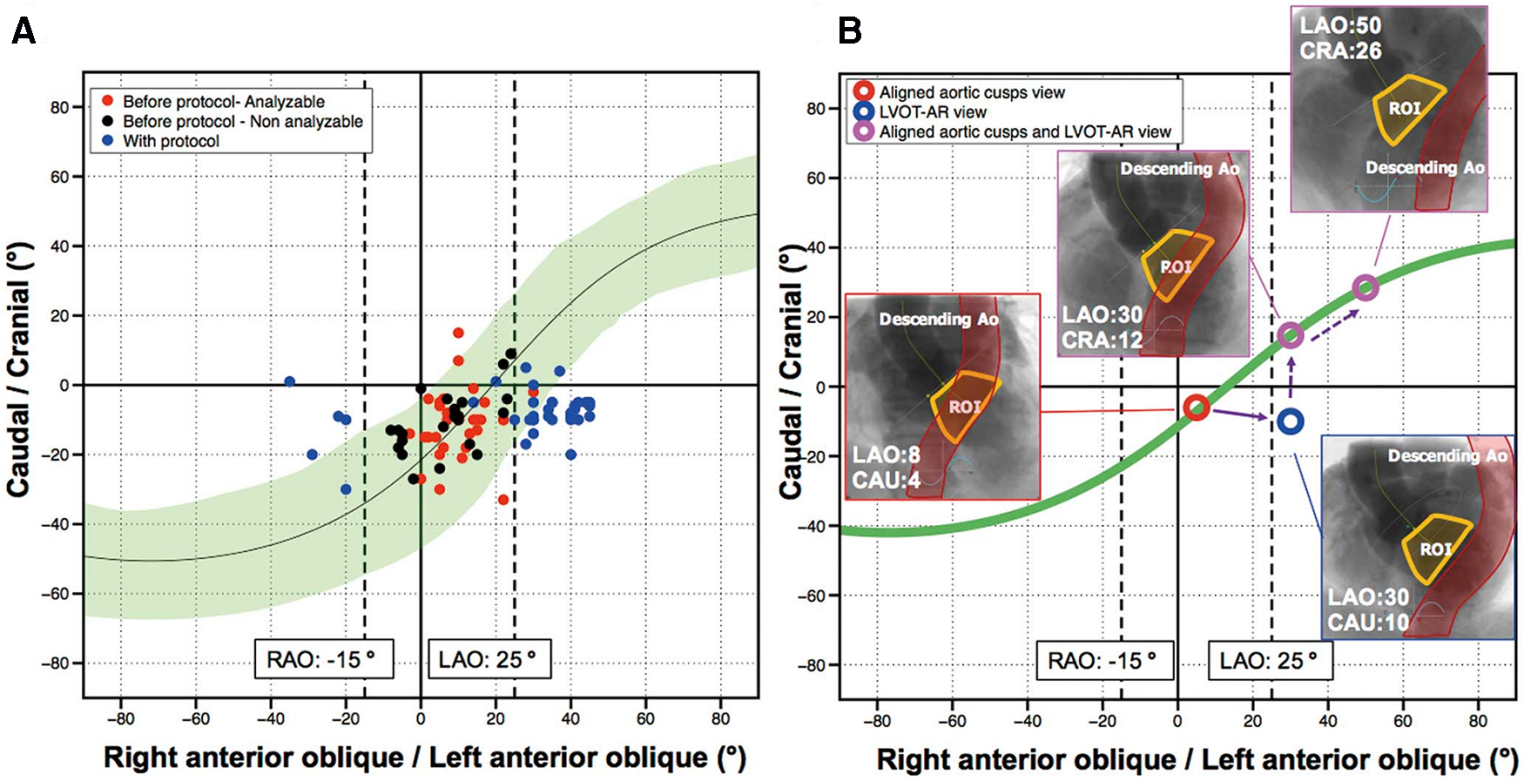
(**A,B**) S curves (mean and 95% C) for optimal acquisition, showing the continuity (S-green curve) of the angiographic projections (*n* = 92) in which the aortic cusps remain aligned and perpendicular to the x-ray beam. One single angiographic view provides optimization of projection for assessment of aortic regurgitation and aligned aortic cusps. Alternative fluoroscopic view with x-ray gantry angled caudally is shown by a blue circle. [Reproduced with permission from Dr Tateishi, et al. (21) and from the Circulation journal].

These promising results paved the way for the ASSESS-REGURGE (NCT03644784) registry, which was a multi-continental trial conducted in four centres in Asia (Japan), North America (Canada), and Europe (the Netherlands and Germany) enrolling 354 consecutive patients with Heart Team consensus in favour of TAVR over a median period of 12 months. In this registry, operators performed the final aortogram according to an angiographic projection which was pre-planned by CT (3mensio or Circle) or visually by fluoroscopy (Teng's rule) (22). An independent core laboratory analysed all images for feasibility and assessed the regurgitation. The acquisition protocol was followed in all 354 patients and all aortograms were analysed by the core lab, with analysis feasible in 95.5% [95% confidence interval (CI): 93.2% to 97.5%] of cases. No differences were observed among the different planning strategies (CT 96.5% vs. Teng's rule 93%, *p* = 0.159: or Circle 98.5% vs. 3mensio 95.8% vs. Teng's rule 93%, *p* = 0.247) (18) Figure 7.

**Figure 8:**
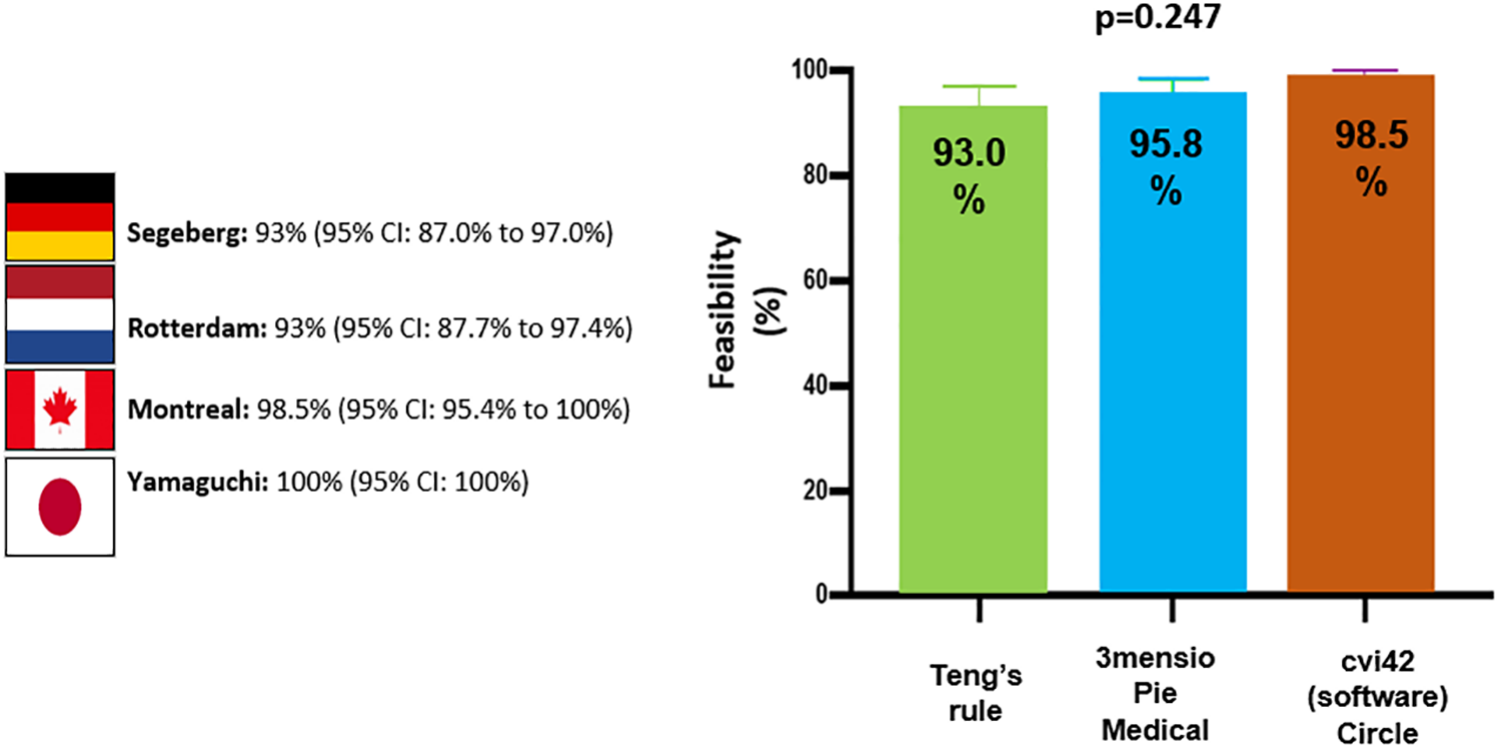
Feasibility of analysis per centre and per planning method [modified from Modolo, et al. (21)].

The next step was to conduct the OVAL trial (Online Video-densitometric Assessment of Aortic Regurgitation in the Cath-Lab; NCT04047082) to determine the feasibility of online assessment of regurgitation (percentage of analyzable cases) and the reproducibility of results between online and offline analysis by the core laboratory. One hundred consecutive patients with aortic stenosis and indications to undergo TAVR were enrolled. The planning of optimal views for video-densitometry was determined with the use of Heart Navigator software (Philips Healthcare, Best, the Netherlands) Supplementary Figure S8. All final aortograms were analyzed with online software in the catheterization laboratory and were also forwarded to an independent core laboratory for blinded offline assessment. The feasibilities of online and offline analysis by the core laboratory were identical (92%; 95% CI:86%–97%, Figure 8). Reproducibility assessment showed a high correlation between online and core laboratory evaluations (*R*^2^ 0.87, *p* < 0.001), with an intraclass correlation coefficient of 0.962 (95% CI: 0.942 to 0.975; *p* < 0.001) (17).

## Comparison of aortic regurgitation following different TAVR devices

**Figure 9:**
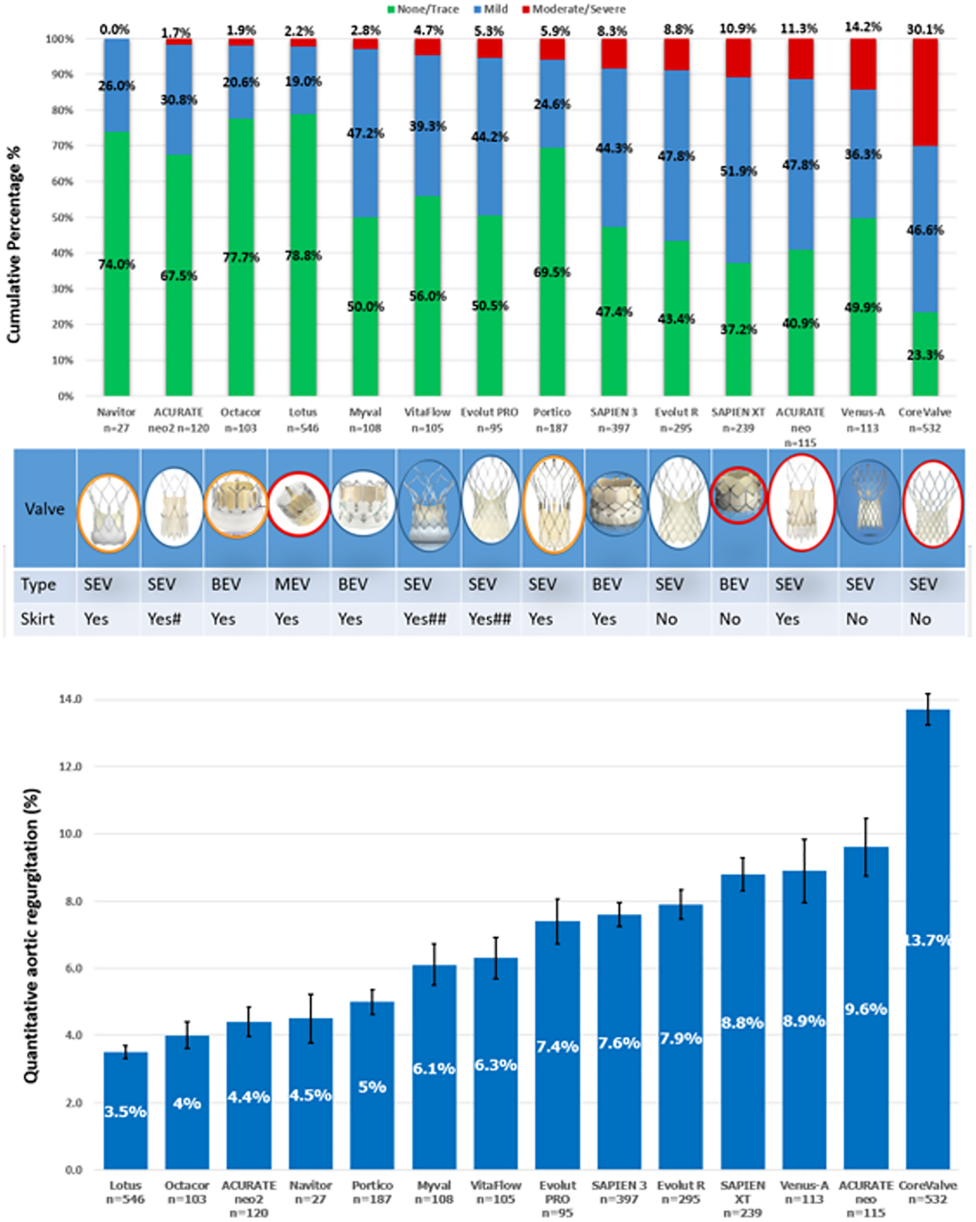
(**A**) Cumulative percentage of different grades of post-TAVR AR by video-densitometric assessment. Devices circled in red are not commercially available anymore. (**B**) Comparison of the mean regurgitation fraction after TAVR among the 14 THVs. Bars denote the mean regurgitation values, and error bars denote standard errors of the mean.

Taking advantage of our large network, we performed a multicentre retrospective corelab pooled analysis of aortograms from over 2,665 consecutive patients treated with 14 different THV devices (23–27) which included first generation devices as well as new generation devices and novel technologies from India and China Figure 9. The results showed that the proportion of patients with moderate or severe regurgitation followed the same ranking order as for RF as a continuous variable. The rates of LVOT-AR > 17% are shown in Figure 9A with the lowest rate 1.7% (ACURATE neo2), and the highest rate 30.1% (CoreValve, *χ*^2^ *p* < 0.001). Figure 9B shows the mean LVOT-AR for each valve with the lowest mean AR 3.5 ± 4.4% (Lotus) and the highest 13.7 ± 10.7% (CoreValve, ANOVA *p* < 0.001).

Post-hoc two-by-two testing showed that the Lotus valve had a significantly lower RF compared with each of the other valves except for the ACURATE neo2, which had a significantly lower RF compared with SAPIEN 3, Evolut R, SAPIEN XT, Venus-A, ACURATE neo, and CoreValve. Similarly, the first generation CoreValve had a significantly higher RF than all the other valves. Apart from the CoreValve, Lotus, and ACURATE neo2, no other valve differed in the amount of RF from each other.

These result highlight that the Lotus valve has the lowest average RF and the ACURATE neo2 the lowest percentage of moderate/severe regurgitation, with the latter device performing significantly better than the ACURATE neo. Myval, VitaFlow and Venus-A are promising options in the THV armamentarium. In our latest report of 103 patients receiving the new Myval Octacor THV, moderate AR was basically eliminated in patients with tricuspid valve morphology (28). Of note, although the incidence of moderate/severe AR has regressed with contemporary THV, the incidence of mild AR remains noticeable, and assessment of its long-term prognosis still requires further investigation to establish whether it has a benign or malignant outcome and whether further refinements (e.g., outside skirt expanding with moisture) of THV will be able to eliminate para valvular regurgitation.

**Figure 10:**
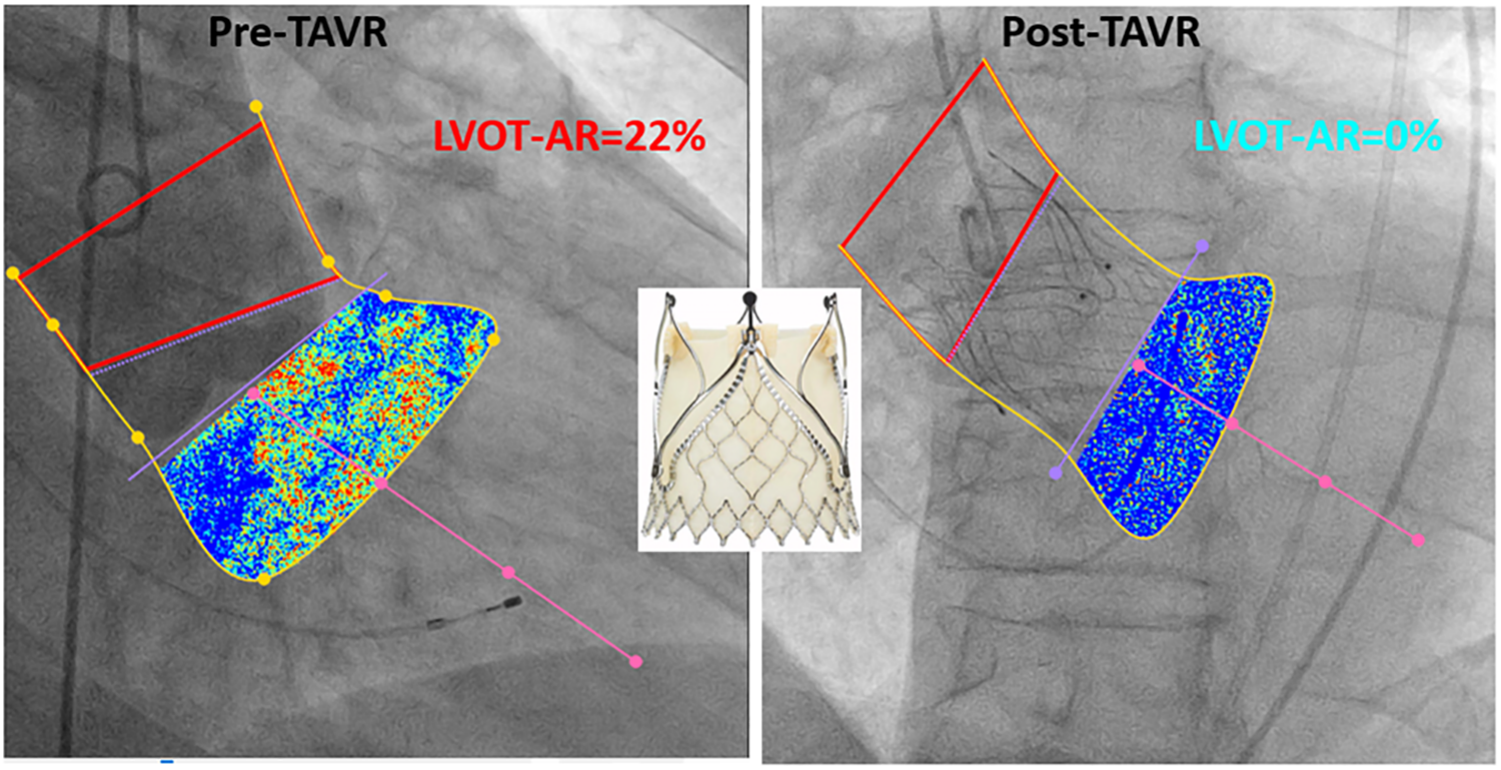
Example of LVOT-AR analysis pre and post TAVR using jenaValve.

With our colleagues and industrial partners, we continue to analyze new commercially available THVs. Analysis post-TAVR using JenaValve (JenaValve Technology, Germany) Figure 10, Portico, Navitor valves (Abbott Structural Heart, St Paul, MN, USA) and Hydra valve (Vascular Innovations Co Ltd, Nonthaburi, Thailand) is ongoing.

Comparison of aortic regurgitation following different TAVR devices by video-densitometry and echocardiography is explained in detail in the Supplementary Data Sheet and Supplementary Figure S9.

## Video-densitometry and evaluation of chronic hemodynamic performance of a bio-restorative THV

Serruys et al. (29) used LVOT-AR software for the evaluation of the long-term hemodynamic performance of different iterations of biorestorative THV in an ovine model, and as shown in Supplementary Figure S10, helped facilitate device development.

## Video-densitometry limitations

Video-densitometric angiography is, however, not without limitations. Various radio-opaque objects intrathoracic or extracorporeal (guidewires, catheters, pacemaker lead, surgical wires, TEE probe, mitral annular calcification, and electrocardiogram electrodes) may interfere with the video-densitometric background of ROI's. Whenever possible the radio-opaque structures not permanently implanted should be removed prior to aortography.

Video-densitometric angiography is also sensitive to pulmonary diaphragm/patient/table motion. If the standardized protocol of acquisition avoiding the over-projection of the ascending and descending aorta over LVOT has dramatically improved the feasibility of analysis (see above), nevertheless it remains that the volume, rate, and time of injection could be further optimized and regulated by single diastolic injection triggered by ECG, thereby reducing the injected amount of contrast medium to a minimum of 8 ml (10, 12). The lack of randomized head-to-head trials comparing video-densitometric angiography and traditional AR assessment is currently a limitation of video-densitometry. However, multiple prospective clinical trials are ongoing to fill the current evidence gap. For example, the multicentre, randomized LANDMARK trial (NCT04275726) will compare the post-TAVR AR of Meril's Myval transcatheter heart valve (THV) series vs. contemporary valve with Video-densitometry assessment and echocardiography.

## Ongoing and future designed trials using video-densitometry for AR assessment

Although still absent on European and American practice guidelines of TAVR, Quantitative aortography with video-densitometric assessment is increasingly recognized as a valid method for aortic regurgitation assessment. Indeed, video-densitometry was mentioned in the VARC III document (30) as an acceptable technique for the evaluation of aortic regurgitation post-TAVR and as such, has been used in ongoing registries and randomized trials (Table 2), e.g., LANDMARK trial (NCT04275726) and Compare TAVR trial (NCT04443023), as well as in the ongoing registries evaluating the ACURATE neo2 (NCT04810195) (24).^.^

**Table 2:** Ongoing and in preparation trials using video-densitometry.

Ongoing trials
LANDMARK RCT (768 patients,74 sites)
Compare TAVI (1,062 patients, 3 sites)
ACURATE neo vs. Ne02 registry (240 patients, 2 sites)
Early ne02 registry (554 patients, 16 sites)
Global ne02 registry (2,000 patients, 87 sites)
PAUSE-VD (100 patients, 2 sites)
Survival with mild AR post TAVR, Bad Krozingen Hospital (464 patients)
In preparation trials
Oval Europe
Oval Japan
Oval China

Currently, the Pause-VD trial (Table 2) investigates the reproducibility in the Cath lab of 2 sequential LVOT-AR performed at a time interval of 10–15 min to investigate the reproducibility shortly after the procedure.

The OVAL GUIDE trials are prospective, multicentre, observational, investigator-initiated studies, aimed at intraprocedural TAVR guidance using online video-densitometric angiographic AR quantification for determining whether a PVL requires further corrective measures. Design of both trial is described in the Supplementary Data Sheet and illustrated in Supplementary Figures S11, S12.

## Future developments

### Mitral video-densitometry

Angiographic assessment of mitral regurgitation fraction (MRF) remains at best semi-quantitative (Sellers's method) and operator dependent. Recently an attempt was made to adapt the video-densitometric methodology to the mitral space. In vitro MRF and MRV were assessed in a mock circulation with transonic flow measurement at a cardiac output comparable as the one measured *in vivo* by thermodilution Supplementary Figure S13C (6).

**Figure 11:**
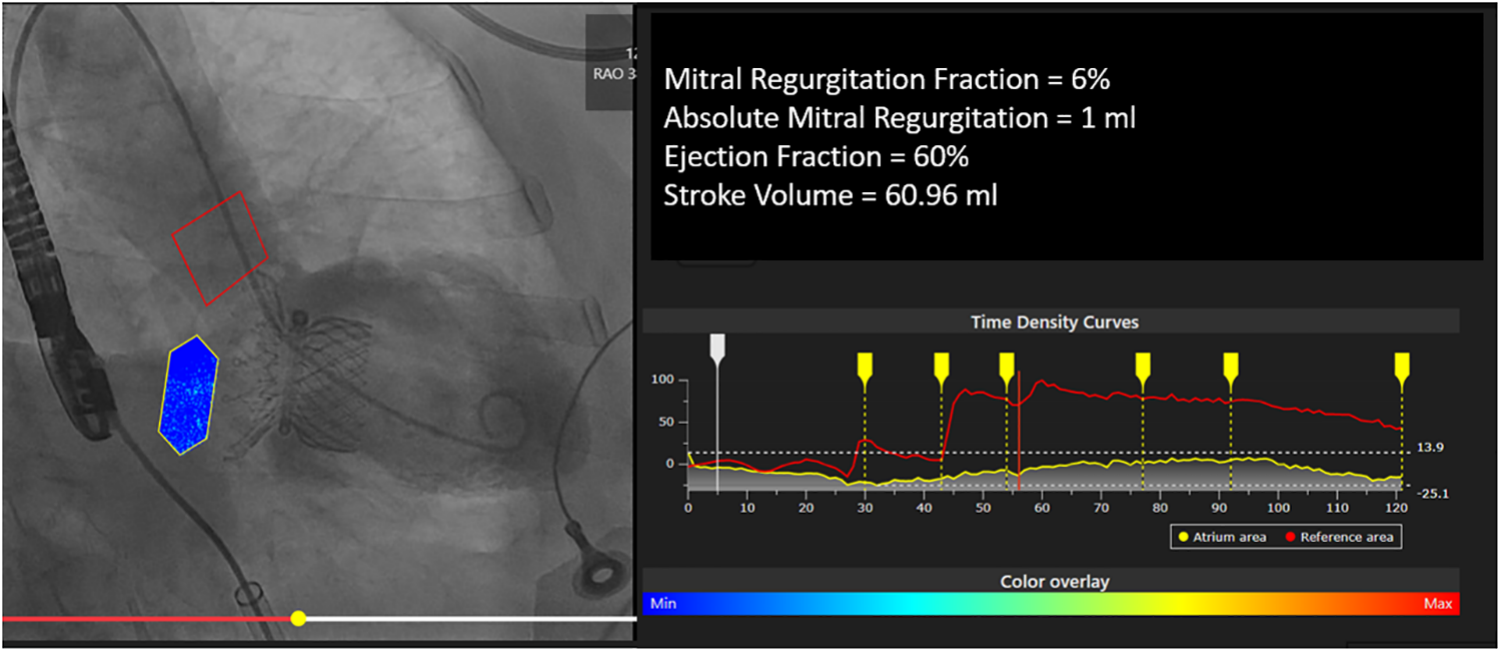
Example of QMR analysis post mitral valve replacement with highlife valve.

The *in vitro* and *in vivo* MRF, MRV, and interobserver reproducibility for QMR analysis strongly correlated. There were also very strong correlations of *in vivo* MRF between 2 independent analysts, Supplementary Figure S13D. It was concluded that *in vivo* MRF using the novel software is feasible, accurate, and highly reproducible. These promising results have led us to initiate the first human feasibility study comprising patients undergoing percutaneous mitral valve edge-to-edge repair (6) or mitral valve replacement Figure 11.

### Three-dimensional video-densitometry

One of the limitations of two-dimensional video-densitometry is the lack of three-dimensional localization of the regurgitation jet. Development of three-dimensional video-densitometric analysis from biplane aortography is ongoing (Supplementary Figure S14).

### Video-densitometry and deep learning

Automatic identification of the aortic root and the LVOT during video-densitometric analysis by applying deep learning algorithms is considered the next step towards a seamless integration of quantitative video-densitometric assessment of aortic regurgitation into a minimalist TAVR approach.

## Conclusions

Quantitative video-densitometric assessment of paravalvular leak by aortography is an objective, accurate, and reproducible tool which has been extensively vetted and validated in-vitro, in-vivo, and in clinical settings, for the assessment of AR following TAVR. Many TAVR devices have been evaluated using video-densitometry, and the technique is currently being used in ongoing trials and registries. Three-dimensional video-densitometric analysis for precise spatial localization of paravalvular jets might be the next frontier.
