# Supplementary material for: Quantitative aortography for assessment of aortic regurgitation in the era of percutaneous aortic valve replacement
Source: Front Cardiovasc Med. 2023 Jul 17;10:1161779. doi: 10.3389/fcvm.2023.1161779 (PMC10389707; doi:10.3389/fcvm.2023.1161779)
Supplement: Supplementary file 1 [file Datasheet1.docx]

**Quantitative aortography for assessment of aortic regurgitation in the era of percutaneous aortic valve replacement.**

**Supplementary Material**

**Word count:** 3837 words

**Video-densitometry Validation of LVOT-AR**

**In silico validation with Mock circulation:**

A mock circulation was used with a 26 mm SAPIEN XT device (Edwards Lifesciences, Irvine, CA, USA) inserted between a silicone tube pressurized with systemic pressure mimicking aortic compliance and a Plexiglas tube connected with a cyclic flow generator simulating LV pressure(7). The Edwards SAPIEN XT valve inserted between the two compartments could be deformed by a radio translucent screw perpendicular to the metallic stent structure of the valve in order to deform it and create increasing levels of PVL **Supplementary Figure 3.** A flow probe (Transonic 28PAU, with TS410 flowmeter; Transonic, Ithaca, NY, USA) was used for the measurement of forward and backward flow (ml/sec). During the manual advancement of the screw the operator watched the increasing RF (%) displayed by the computer of the mock circulation **Supplementary Figure 3.** At different pre-set levels of regurgitation, aortograms were cinefilmed and performed with various volumes, pressure, and speed of injections, using either asynchronized injections (injection took place at a random time-point in the cardiac cycle) or injection, triggered and synchronized with a single diastolic phase.

A substantial correlation between video-densitometric parameters (LV-AR, LVOT-AR, qRA index, relative max, and maximum upslope) and RF (r^2^=0.96, 0.96, 0.93, 0.87, and 0.93; p<0.001 for all) was seen. LV-AR (ROI=entire LV) and LVOT-AR (ROI=LVOT) were similar (p=0.51) and strongly correlated (r^2^=0.99) with a mean difference of 1.92% (95% limits of agreement: ±2.83). The correlations of LV-AR and LVOT-AR with RF were stronger when more than one cardiac cycle was included in the analysis (one cycle: r^2^=0.85 and r^2^=0.83; four cycles: r^2^=0.96 and r^2^=0.96, for LV-AR and LVOT-AR, respectively), however including more than four cycles did not improve accuracy further(13). In that experimental setting the positioning of the Pigtail catheter in the aorta with respect to the leaflets of the bio-prosthesis was also investigated; there was no variation in AR as determined by video-densitometry provided the tip of the catheter was kept 20-40mm from the level of the leaflets in annular and supra-annular trans-catheter heart valve (THV) systems.

**In vivo validation in porcine models**

In an anesthetized pig, increasing levels of central (transvalvular) AR were induced by inserting long (80 mm) femoral self-expanding stents (Wallstents, Boston Scientific) of increasing diameters (6, 7, 8, and 10 mm) deployed into the aortic valve to prevent coaptation of the leaflets and to induce AR of various severities. The stents were inserted over a 0.035" Safari^2^™ guide wire (Boston Scientific, Marlborough, Massachusetts, USA), and were partially deployed so that they could be retrieved and exchanged with stents of larger diameter. The unconstrained diameters of the stents, as well as their 2D angiographic contours as assessed by QCA were correlated with the video-densitometric assessment of the regurgitation. There was a significant correlation between the RF assessed by LVOT-AR (28%, 40%, 49%, and 58%) and the Wallstents sizes (6, 7, 8, and 10 mm, P=0.016) as well as with the area of non-coaptation measured by QCA (r = 0.822, P < 0.001) (8) **Supplementary Figure 4.**

**Validation of synchronized injection to minimize the amount of contrast**

Miyazaki et al.(12) used an ACIST CVi® contrast delivery system (ACIST Medical Systems, Eden Prairie, MN, USA) for contrast injection in the mock circulation described in **Supplementary** **Figure 3**. For the synchronised diastolic injection, the injector was triggered 490 msec before the start of diastole by a “servomotor” signal from the mock LV pump. Quantitative video-densitometric measurements of PVL were performed in non-synchronised and synchronized injections. The volume of contrast required for the synchronised diastolic injection was significantly less than in the non-synchronised injection (8.1 [7.9-8.5] ml vs. 19.4 [19.2-19.9] ml, p<0.001). The correlation between the two methods was high (Spearman’s coefficient rho ranging from 0.991 to 0.968). Intra observer intra-class correlation coefficient for both methods of injection was 0.999 (95% CI: 0.996-1.000) for the synchronised diastolic and 0.999 (95% CI: 0.996-1.000) for the non-synchronised injection group. The mean difference between the two modes of injection was 0.17% and limits of agreement were ±1.64% **Supplementary Figure 5.**

In the porcine model with the self-expanding stent deployed across the valve (see above) Modolo et al.(10) confirmed that a synchronized diastolic injection triggered by the QRS complex of the animal with low contrast volume (8 ml) produced paradoxically denser images in the aortic root and provided more radiopaque aortograms than the conventional asynchronous injections of a larger volume of contrast (p=0.04 for density). The regression line between X and Y performed with the synchronized synchronous injection/low volume resulted in a lower intercept and a slope closer to the line of identity (y = 11.9 + 0.79x, p<0.001, r^2^=0.94) than the intercept and slope obtained with non-synchronous injections of 8mL or 15mL (y=26.5 + 0.55x, p<0.001, r^2^=0.81). It is unfortunate that manufacturers involved with the technology of pump injectors have not yet fully appreciated as an unmet need in TAVR procedures the clinical value of a greatly reduced volume of contrast medium, administered during a diastolic aortogram triggered by the QRS complex and strictly synchronic synchronised to a single period of diastole **Supplementary Figure 6.**

**Correlation of LVOT-AR with Cardiac Magnetic Resonance post TAVR:**

In 2018, Abdel-Wahab et al. compared LVOT-AR by video-densitometry to CMR derived regurgitation fraction (CMR-RF) for the quantification of PVL in 135 patients after TAVR. The average CMR-RF was 6.7±7.0% whereas the average LVOT-AR was 7.0±7.0%; with a substantial correlation (r=0.78, p<0.001). On receiver-operating characteristic curves, an LVOT-AR ≥10% corresponded to >mild PVL as defined by CMR-RF (AUC: 0.94; p<0.001; sensitivity 100%, specificity 83%), whereas an LVOT-AR ≥25% corresponded to moderate-to severe PVL (AUC: 0.99; p<0.004; sensitivity 100%, specificity 98%). Intra-observer reproducibility was excellent for both techniques (CMR-RF, intraclass correlation coefficient: 0.91, p<0.001; LVOT-AR intraclass correlation coefficient: 0.93, p<0.001). These results confirm that LVOT-AR provides a surrogate assessment of PVL severity after TAVR that correlates well with the CMR-RF **Supplementary Figure 7.**

**Aortic regurgitation index (ARI) and video-densitometry**

In a healthy pig, we analysed sequentially different levels of regurgitation by LVOT-AR (see above) and compared with ARI recorded with a double tip manometer catheter. LVOT-AR/ARI in the healthy animal was correlated. However, in patients with alteration in left ventricular and aortic compliance, the Bad Krozingen group did not find a significant correlation between ARI and echocardiographic regurgitation and could not confirm a long-term prognostic value of ARI, at variance with the echocardiographic assessment of AR that predicts mortality long term (9).

**Comparison of aortic regurgitation following different TAVR devices by video-densitometry and echocardiography**

Our findings show higher proportions of moderate/severe AR than those reported with echocardiography(10-19) **Supplementary Figure 9**, which is concordant with the results of the CHOICE trial(20), wherein the percentage of more than mild AR was 18.1% for self-expanding and 4.1% for balloon-expandable THVs by angiography in comparison to 5.8% and 1.6% by echocardiography, respectively. There are several possible reasons for these differences; first, the patient cohorts in these trials were selected according to detailed inclusion/exclusion criteria in contrast to our pooled analysis cohort, which was real-world all-comers. Second, PVL assessment is traditionally detected, and semi quantified by echocardiography, however, this modality of examination is not completely exempt from limitations e.g. assessment of AR is significantly influenced by the imaging plane, with low sensitivity in detecting posterior jets in the short axis view(1), whilst significant variability also exists in the reported incidence of “what is mild” versus “what is moderate” among different core labs (Kappa= 0.481 and 0.517 for PVL class 4 and PVL class 7 grading, respectively)(2). Thirdly, in our study we assessed immediate regurgitation (within seconds to minutes of implantation), as opposed to echocardiographic assessment, which is usually performed at discharge or 30-day post-TAVR (21,22). On the other hand, the sealing feature of the anti-leak skirts of novel devices may take minutes to achieve their complete functionality. Thus, deferred echocardiography may assess regurgitation with a fully operating anti-leak skirt, with a consequently lower amount of regurgitation. However, we reported a good correlation between the AR measured immediately post-procedure using video-densitometric assessment and by CMR performed on average 10 days post-procedure(23). In addition, in two cohorts of patients, we correlated the LVOT-AR assessed immediately post-procedure with trans-thoracic echocardiography pre-discharge, and showed that the categorical degree of regurgitation was concordant between the two(23). Moreover, data on the late reduction or increase of paravalvular leakage is inconsistent in the literature(24). In BEVs THVs, recoil could be a potential factor for the late increase, whereas with SEVs, late self-expansion could explain the reduction in PVL. In addition to these opposing effects, the internal and external skirt (potentially absorbing fluid) may also affect the progression in PVL over time.

**OVAL GUIDE trials design**

While the minimalist TAVR approach is nowadays the norm in Europe, Asian countries (Japan and China), are still performing TAVR procedures under general anaesthesia and with TEE guidance. Three trials, OVAL GUIDE Europe, Japan and China have been designed. OVAL Europe is a triple blinded registry comparing operator decision based on visual Seller versus online video-densitometric analysis by a technician in the interventional suite versus offline retrospective analysis in the Core lab **Supplementary Figure 11**. OVAL Japan and China will be a randomized trial comparing the decision regarding BPD guided by either TEE guidance in patients under general anaesthesia or video-densitometric analysis in patients under sedation **Supplementary Figure 12**.

**Mitral video-densitometry**

Fourteen sheep following surgical mitral valve replacement using either a commercial bio-prothesis or an experimental polymeric prosthesis were catheterized, cardiac output was measured by thermodilution and a left ventriculogram was acquired to assess the MFR using novel software adjusted to the mitral space, prior to euthanasia carried out at predefined times of follow up. All valves with surrounding tissue were explanted, mounted into a valve holder of a mock-circulation to test the performances of the explanted Valve in-vitro. The *in-vitro* MRF and Mitral Regurgitation volume (MRV) parameters assessed in the mock circulation with transonic flow measurements, calculated a cardiac output comparable with the measurement obtained by thermodilution during the catheterization.

The principle of the qMR software (CAAS QMR 0.1 Pie Medical, Maastricht, the Netherlands) is to use a left ventriculogram to quantify the time-density AUC from the left atrium (ROI, representative of MR) and a reference area at the aortic root (reference area [RA], representative of aortic forward flow) **Supplementary Figure 13**. The ROI in the LA is a transit zone of regurgitation with a hexagonal shape to cover eccentric jets. The *in-vivo* derived MRF by QMR can be calculated from the following formula:

$$In-vivo derived MRF (\%)=\left( \frac{{AUC}_{ROI (LA)}}{{AUC}_{ROI (LA)}+{AUC}_{ROI (aortic root)}} \right)*100$$

In this formula, the time-density AUC in the aortic root is a surrogate of the aortic forward flow volume (AFFV) and the time-density AUC in the LA a surrogate of MRV, with the sum of both a surrogate of stroke volume (SV) **Supplementary Figure 13B**. Left ventricular volumetric analysis was used to compute SV in ml and to convert MRF into MRV. Assuming no aortic regurgitation, we can exemplify the formula in a clinical case as follows: if a patient has an end-diastolic volume (EDV) of 240 ml and an end-systolic volume (ESV) of 140 ml, his SV amounts to 100 ml. If 40 ml flow toward the aorta (AFFV) and 60 ml flow backward into the LA (MRV, that is MR), MRF amounts to 60% (60 ml/100 ml) **Supplementary Figure 13A**.

References

1. Abdelghani M, Soliman OI, Schultz C, Vahanian A, Serruys PW. Adjudicating paravalvular leaks of transcatheter aortic valves: a critical appraisal. Eur Heart J 2016;37:2627-44.

2. Hahn RT, Pibarot P, Weissman NJ, Rodriguez L, Jaber WA. Assessment of paravalvular aortic regurgitation after transcatheter aortic valve replacement: intra-core laboratory variability. J Am Soc Echocardiogr 2015;28:415-22.

3. Mihara H, Shibayama K, Jilaihawi H et al. Assessment of Post-Procedural Aortic Regurgitation After TAVR: An Intraprocedural TEE Study. JACC Cardiovasc Imaging 2015;8:993-1003.

4. Sherif MA, Abdel-Wahab M, Beurich HW et al. Haemodynamic evaluation of aortic regurgitation after transcatheter aortic valve implantation using cardiovascular magnetic resonance. EuroIntervention 2011;7:57-63.

5. Ribeiro HB, Le Ven F, Larose E et al. Cardiac magnetic resonance versus transthoracic echocardiography for the assessment and quantification of aortic regurgitation in patients undergoing transcatheter aortic valve implantation. Heart 2014;100:1924-32.

6. Abdelghani M, Tateishi H, Spitzer E et al. Echocardiographic and angiographic assessment of paravalvular regurgitation after TAVI: optimizing inter-technique reproducibility. Eur Heart J Cardiovasc Imaging 2016;17:852-60.

7. Abdelghani M, Miyazaki Y, de Boer ES et al. Videodensitometric quantification of paravalvular regurgitation of a transcatheter aortic valve: in vitro validation. EuroIntervention 2018;13:1527-1535.

8. Modolo R, Miyazaki Y, Chang CC et al. Feasibility study of a synchronized diastolic injection with low contrast volume for proper quantitative assessment of aortic regurgitation in porcine models. Catheterization and cardiovascular interventions : official journal of the Society for Cardiac Angiography & Interventions 2019;93:963-970.

9. Schoechlin S, Hein M, Brennemann T et al. 5-Year outcomes after transcatheter aortic valve implantation: Focus on paravalvular leakage assessed by echocardiography and hemodynamic parameters. Catheterization and cardiovascular interventions : official journal of the Society for Cardiac Angiography & Interventions 2022.

10. Adams DH, Popma JJ, Reardon MJ et al. Transcatheter Aortic-Valve Replacement with a Self-Expanding Prosthesis. New England Journal of Medicine 2014;370:1790-1798.

11. Choudhury T, Solomonica A, Bagur R. The Evolut R and Evolut PRO transcatheter aortic valve systems. Expert Review of Medical Devices 2019;16:3-9.

12. Delgado-Arana JR, Gordillo-Monge MX, Halim J et al. Early clinical and haemodynamic matched comparison of balloon-expandable valves. Heart 2021.

13. Falk V, Wöhrle J, Hildick-Smith D et al. Safety and efficacy of a repositionable and fully retrievable aortic valve used in routine clinical practice: the RESPOND Study. Eur Heart J 2017;38:3359-3366.

14. Leon MB, Smith CR, Mack MJ et al. Transcatheter or Surgical Aortic-Valve Replacement in Intermediate-Risk Patients. New England Journal of Medicine 2016;374:1609-1620.

15. Mack MJ, Leon MB, Thourani VH et al. Transcatheter Aortic-Valve Replacement with a Balloon-Expandable Valve in Low-Risk Patients. New England Journal of Medicine 2019;380:1695-1705.

16. Ruck A. Results from the Early neo2 Registry Acurate neo2 TAVI Valve. Presented at the Congress for the European Association of Percutaneous Cardiovascular Interventions (EuroPCR); 2021. Available online: <https://media.pcronline.com/diapos/EuroPCR2021/3890-20210518_0922_Clinical_Science_Ruck_Andreas_0000_(7805)/Ruck_Andreas_20211805_1408_VOD.pdf> (accessed on 6 Feb 2021).

17. Song G, Jilaihawi H, Wang M et al. Severe Symptomatic Bicuspid and Tricuspid Aortic Stenosis in China: Characteristics and Outcomes of Transcatheter Aortic Valve Replacement with the Venus-A Valve. Structural Heart 2018;2:60-68.

18. Tamburino C, Bleiziffer S, Thiele H et al. Comparison of Self-Expanding Bioprostheses for Transcatheter Aortic Valve Replacement in Patients With Symptomatic Severe Aortic Stenosis. Circulation 2020;142:2431-2442.

19. Zhou D, Pan W, Wang J et al. VitaFlow™ transcatheter valve system in the treatment of severe aortic stenosis: One‐year results of a multicenter study. Catheterization and Cardiovascular Interventions 2020;95:332-338.

20. Abdel-Wahab M, Mehilli J, Frerker C et al. Comparison of Balloon-Expandable vs Self-expandable Valves in Patients Undergoing Transcatheter Aortic Valve Replacement: The CHOICE Randomized Clinical Trial. JAMA 2014;311:1503-1514.

21. Mack MJ, Leon MB, Thourani VH et al. Transcatheter Aortic-Valve Replacement with a Balloon-Expandable Valve in Low-Risk Patients. N Engl J Med 2019;380:1695-1705.

22. Popma JJ, Deeb GM, Yakubov SJ et al. Transcatheter Aortic-Valve Replacement with a Self-Expanding Valve in Low-Risk Patients. New England Journal of Medicine 2019;380:1706-1715.

23. Abdel-Wahab M, Abdelghani M, Miyazaki Y et al. A Novel Angiographic Quantification of Aortic Regurgitation After TAVR Provides an Accurate Estimation of Regurgitation Fraction Derived From Cardiac Magnetic Resonance Imaging. JACC Cardiovascular interventions 2018;11:287-297.

24. Douglas PS, Hahn RT. Echocardiography in Clinical Trials for TAVR: Getting to the Core of the Matter∗. JACC: Cardiovascular Imaging 2015;8:1376-1378.

**Supplementary Figure 1: Adjudicating paravalvular leaks using echocardiography: a critical appraisal.**

**(A)** The assessment of AR is significantly influenced by the imaging plane selected by the examiner with the upper and lower panels showing variability of PVL according to two minimally different imaging planes. An imaging plane that is too low/too high or tangential can lead to a false-negative or overestimation(1)

**(B)** In the transverse short-axis view there is a low sensitivity to detect jets in certain locations (mainly for posterior jets)(1) with the lower panel showing the detection of jets varies from 35% in the 2 o’clock position to 0% in the 7 o’clock position.

**(C)** From a feasibility perspective, even when performed under core lab proctoring, up to 13% of post-TAVR echocardiograms are of inadequate quality to be reliably adjudicated according to the VARC-recommended multi-parametric approach(2). Furthermore, there is significant variability in the reported incidence of “what is mild” versus “what is moderate” among different core labs (Kappa= 0.481 and 0.517 for PVL 4 -class and 7-class PVL grading respectively)(2)**.**

**(D)** Inter-modality inconsistency: in contrast to the good agreement between echocardiographic, angiographic, and cardiac MRI (CMR) assessment of native AR and surgical prosthetic AR, the agreement on PVL grade between echocardiographic and angiographic assessment post-TAVR is achieved in only 56% of cases (inter-technique kappa, 0.14–0.20)(3,4). Similarly, the grading of PVL on echocardiography is often at odds with CMR assessment (inter-technique kappa, 0.20)(4). The severity of native AR as determined by the multiparametric trans-thoracic echocardiogram (TTE) approach correlated well with the regurgitant volume and regurgitant fraction determined by CMR prior to TAVR (Spearman’s rank correlation coefficient [Rs]) =0.79 and 0.80, respectively; p<0.001 for both). However, after TAVR, the correlation between the severity of prosthetic AR assessed by TTE and the regurgitant volume and fraction measured by CMR was only modest (Rs=0.59 and 0.59, respectively; p<0.001 for both), with the severity of AR underestimated by TTE in 61.9% of patients (1 grade in 59.5%). The TTE jet diameter in the parasternal view and the multiparametric approach (Rs) =0.62 and 0.59, respectively; both with p<0.001) had the best correlation with CMR regurgitant fraction post-TAVR. The circumferential extent of prosthetic paravalvular regurgitation on echocardiography correlated poorly with CMR regurgitant volume and fraction (Rs=0.32, p=0.084; Rs=0.36, p=0.054, respectively)(5). These observations underscore the fact that the extrapolation of the diagnostic accuracy of native or surgical prosthetic AR to post-TAVR AR might not always be warranted(1). (compiled from Mihara, et al. (3); Abdelghani et al.(6); Hahn, et al.(2) and Ribeiro HB, et al.(5)).

**Supplementary Figure 2 A)** single frame from digital subtraction aortogram in a patient with 1+ aortic regurgitation by cineaortography. **B)** Summation of density within the aortic (Ao) and left ventricular (LV) areas of interest Digital subtraction aortography besides conventional aortography was used and the ratio in density of left ventricular/aortic area were calculated in each frame and then plotted versus time (time-density curves) The ratio at the end of injection (LV_d_/Ao_d_) had an excellent correlation with visual assessment on cine aortography (Sellers analysis with 4 grades, χ²=19, p <0.001), ranging from 0 to 0.2 in patients with no AR, 0.2 to 0.5 in those with 1+ AR, 0.5 to 0.7 in those with 2+ AR, 0.7 to 0.9 in those with 3+ AR and >0.9 in those with 4+ AR (modified from Klein, et al.(4) with permission from Am J Cardiol).

**Supplementary Figure 3 A)** Mock circulation system and experimental setting. (See text) **B)** Flow rate curves and reverse flow measured by a transonic probe (on the right side) and (on the left side) video-densitometric analysis showing the aortogram, the colour coded cumulative assessment of density and the time density curves (red curve corresponding to the aortic area of reference and yellow curve corresponding to the LVOT area of regurgitation (modified from Abdelghani, et al.(30)).

**Figure 4.** Correlation between video-densitometric regurgitation fraction (VD RF) and transonic flow assessment of reverse flow and hemodynamic Regurgitation Fraction for both injection techniques: synchronized and asynchronized. (modified from Miyazaki, et al.(12)).

**Supplementary Figure 5 A)** Schematic representation of aortic regurgitations of different severity induced by deployment of self-expanding stents of different diameters. **B)** and **C)** partial deployment of progressively larger Wallstents (6, 7, 8, and 10 mm). **D)** Correlations between quantitative AR (VD-AR %) assessment and different unconstrained Wallstent sizes (modified from Modolo, et al. (13)).

**Supplementary Figure 6A)**, **B)** Synchronized and non-synchronized injections in animal model. Coloured curves show density variations during systole and diastole. Red curve represents the mean video-density following a synchronized diastolic injection triggered by the QRS complex. Contrast medium selectively injected during diastole with peak density reached just prior the ejection phase. Blue curve represents a non-synchronized, starting in the ejection phase and reaching a lower peak of density prior to the ejection phase. **C)** Passing-Bablock regression of VD-AR results between SYNC (8ml) and NS (15 ml) injections.

**Supplementary Figure 7A)** (Top, left) post TAVR aortogram with colour coded video-densitometric analysis and examples of time density curves (Top, right) cardiac magnetic resonance (CMR) image with examples of regurgitation fraction (RF) (CMR-RF) (Middle and Bottom). **B)** cumulative curves of CMR-RF and LVOT-AR and Linear Correlation (modified from Abdel-Wahab, et al.(16)).

**Supplementary Figure 8 A)** online planning of aortography with the use of Heart Navigator software (left screen) side by side with the online software of LVOT-AR (right screen) **B)** graphic description of analysability of 100 patients, (modified from Modolo, et al. (23)).

**Supplementary Figure 9.** Paravalvular regurgitation (%) as reported by echocardiography (discharge or 30 days) in the main TAVR trials.

**Supplementary Figure 10.** Five design iterations (A, B, B’, C, and D) of the Xeltis BTHV (Xeltis BV, Eindhoven, the Netherlands) were implanted transapically in 46 sheep with long-term serial data available in 39 animals. Hemodynamic assessment was performed at implantation, 3-, 6-, and 12-months including quantitative aortography, echocardiography, and histology. Aortography was performed according to a pre-specified protocol immediately after TAVR (acute phase) and before scheduled euthanasia (chronic phase). The mean VD-AR at implantation, 3, 6, and 12 months among the 5 design iterations are shown here with only iterations A and D having a mean RF below the critical threshold of 17%, and in conjunction with echocardiographic findings and histologic data these video-densitometric data have facilitated strategic decisions regarding further device development (modified from Serruys, et al.(14)).

**Supplementary Figure 11.** OVAL Europe study design.

**Supplementary Figure 12.** OVAL Japan study design.

**Supplementary Figure 13. A)** In presence of MR, the flow from the LV (stroke volume [SV], ml) is divided into LV to aorta (aorta forward flow volume [AFFV], ml) and LV to LA (MRV, ml). The ratio of MR (%) = MRV (ml)/SV (ml). SV (ml) = end-diastolic volume (EDV) (ml) - end-systolic volume (ESV) (ml). MFFV (ml) is the forward flow into the LV through the mitral valve, which is equal to the stroke volume. **B)** use of a left ventriculogram to quantify the time-density area under the curves (AUCs) from the LA (region of interest [ROI], representative of mitral regurgitation [MR]) and the aortic root (reference area [RA], representative of aortic forward flow). **C)** explanted valves tested in a mock circulation. **D)** corelation between in vitro derived MRF versus in vivo derived MRF by angiographic QMR of 3 beats (modified from Kawashima, et al. (6)).

**Supplementary Figures**

**Supplementary Figure 1**

**
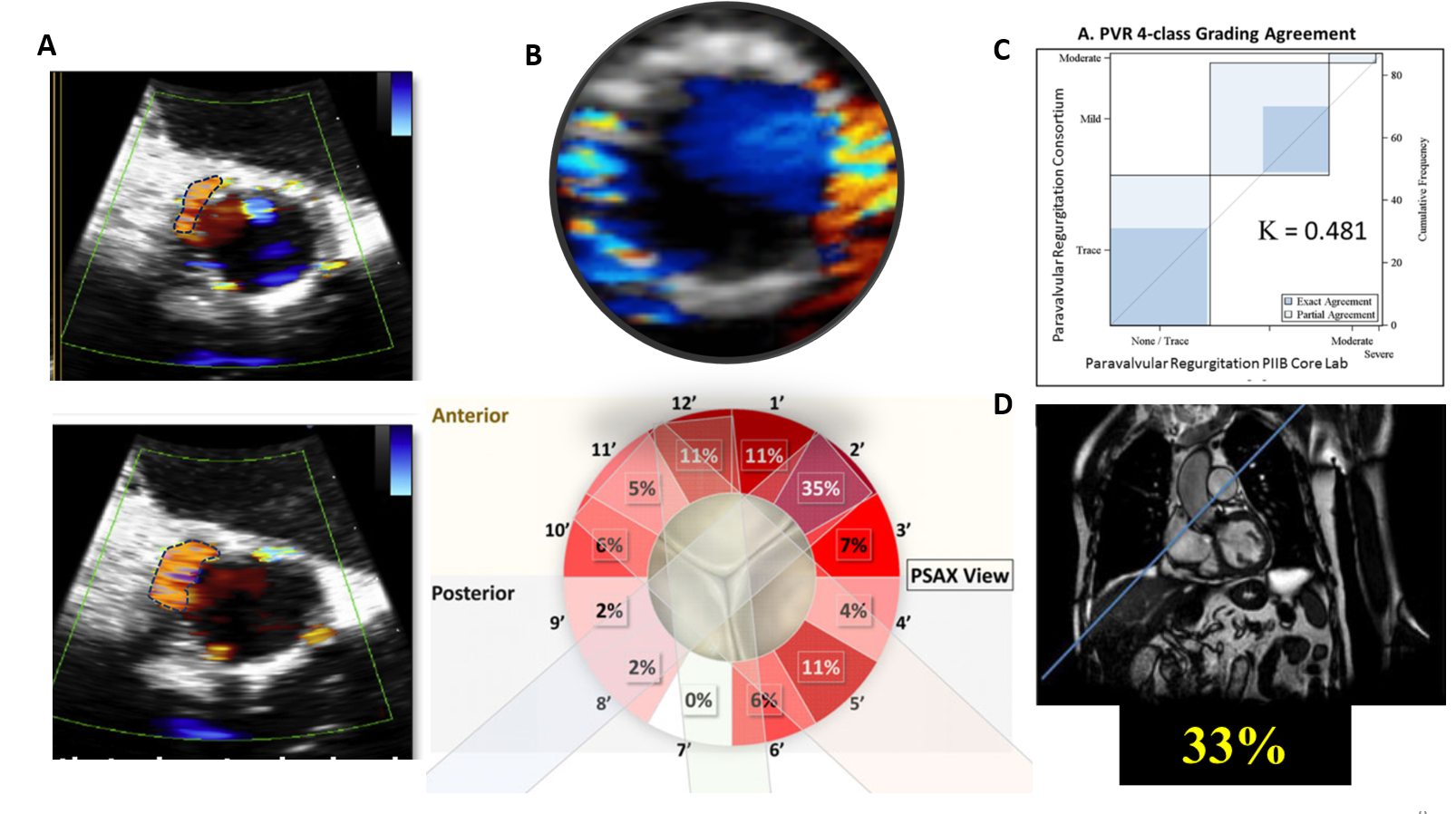
**

**Supplementary Figure 2**


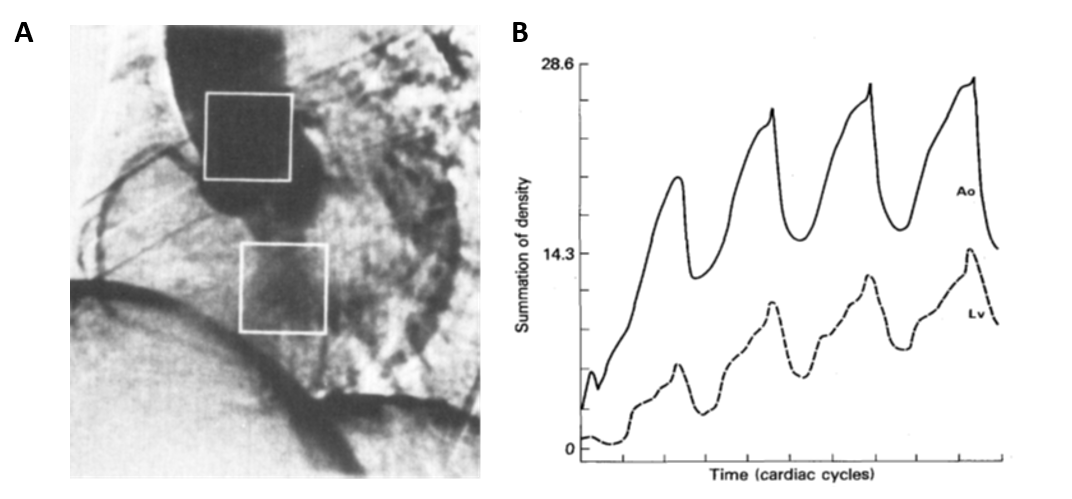


**Supplementary Figure 3**


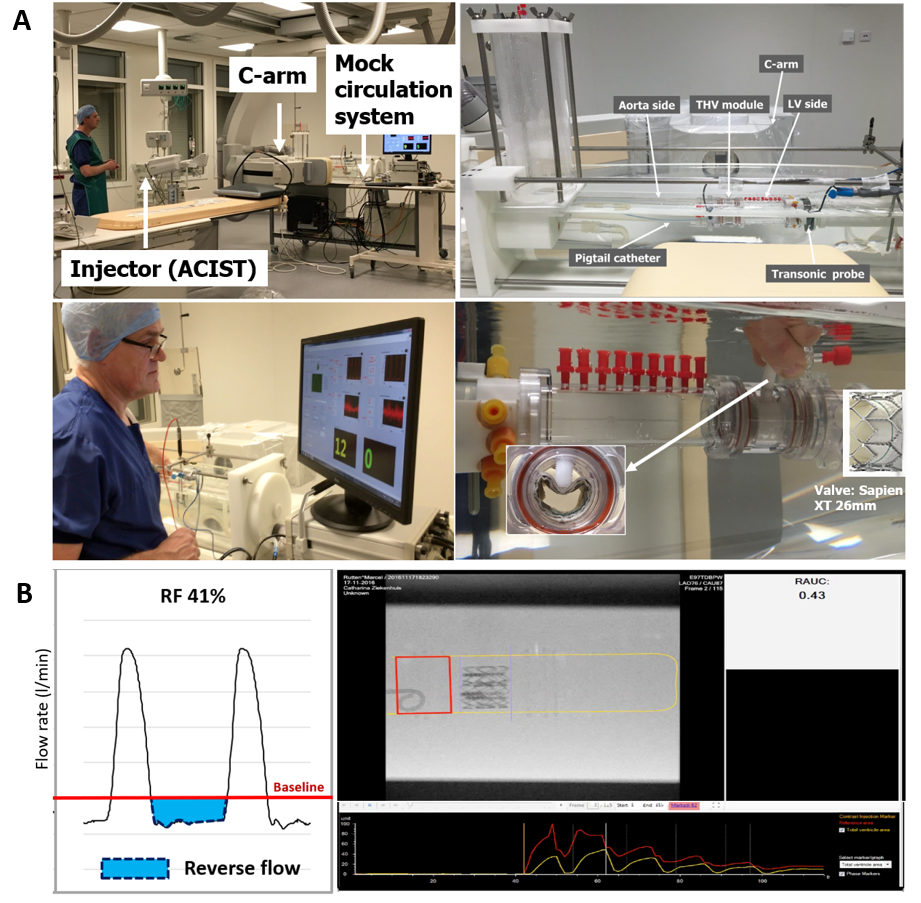


**Supplementary Figure 4**


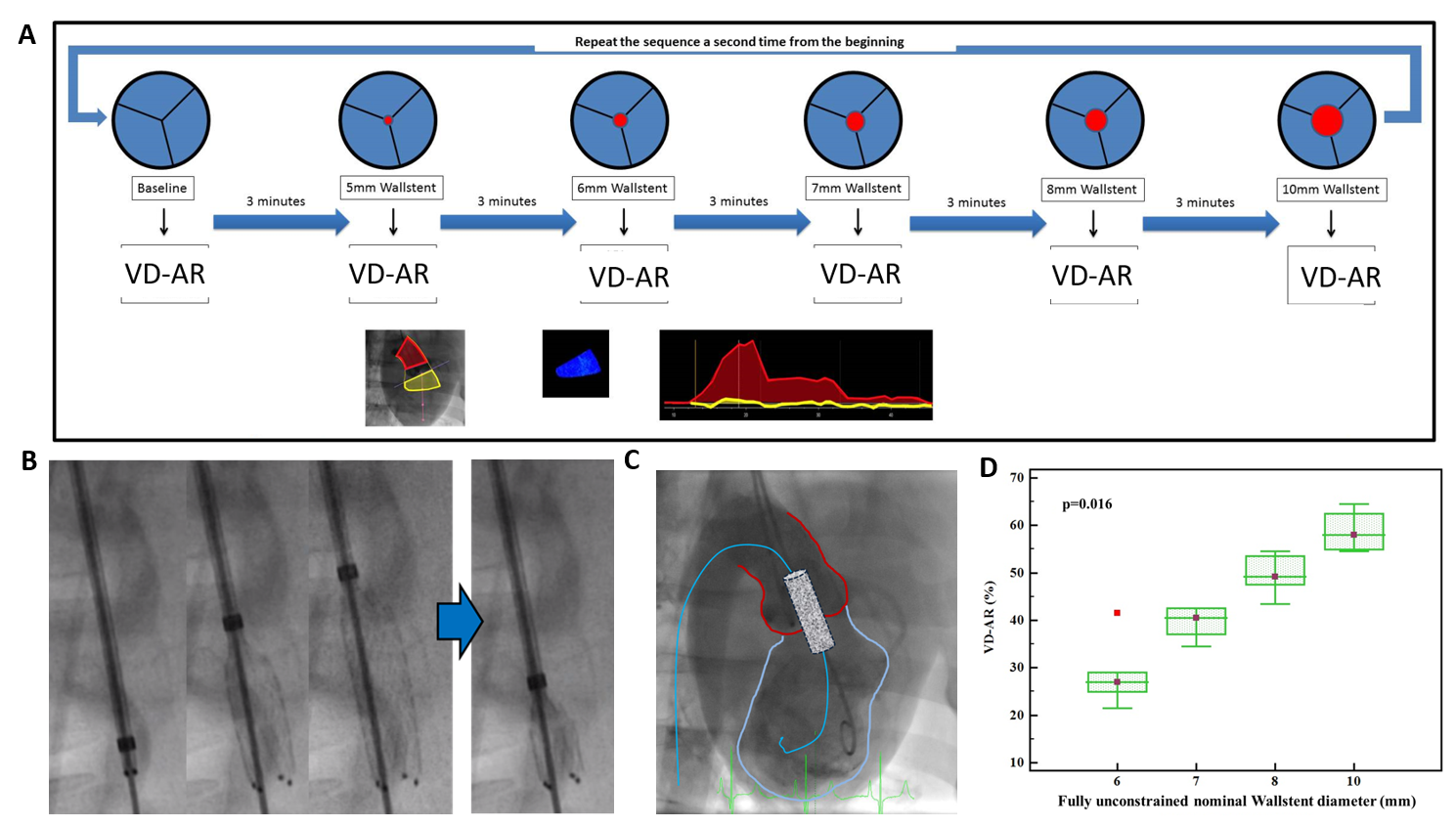


**Supplementary Figure 5**


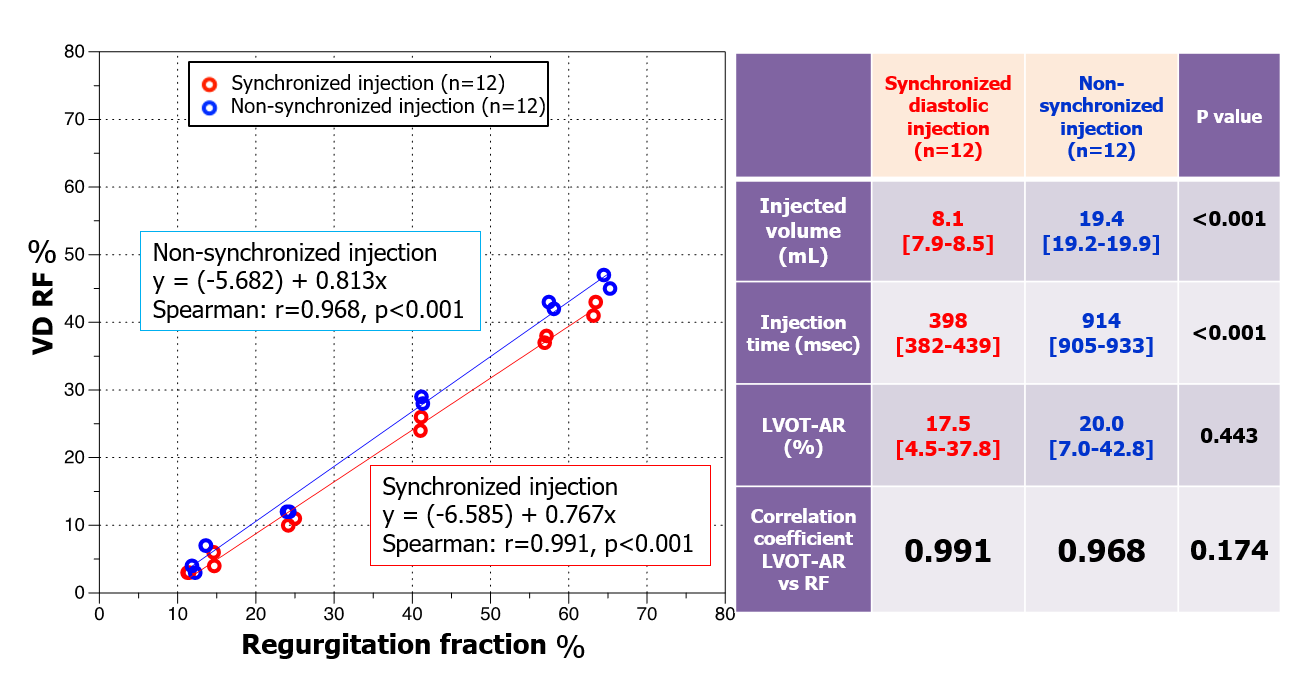


**Supplementary Figure 6**


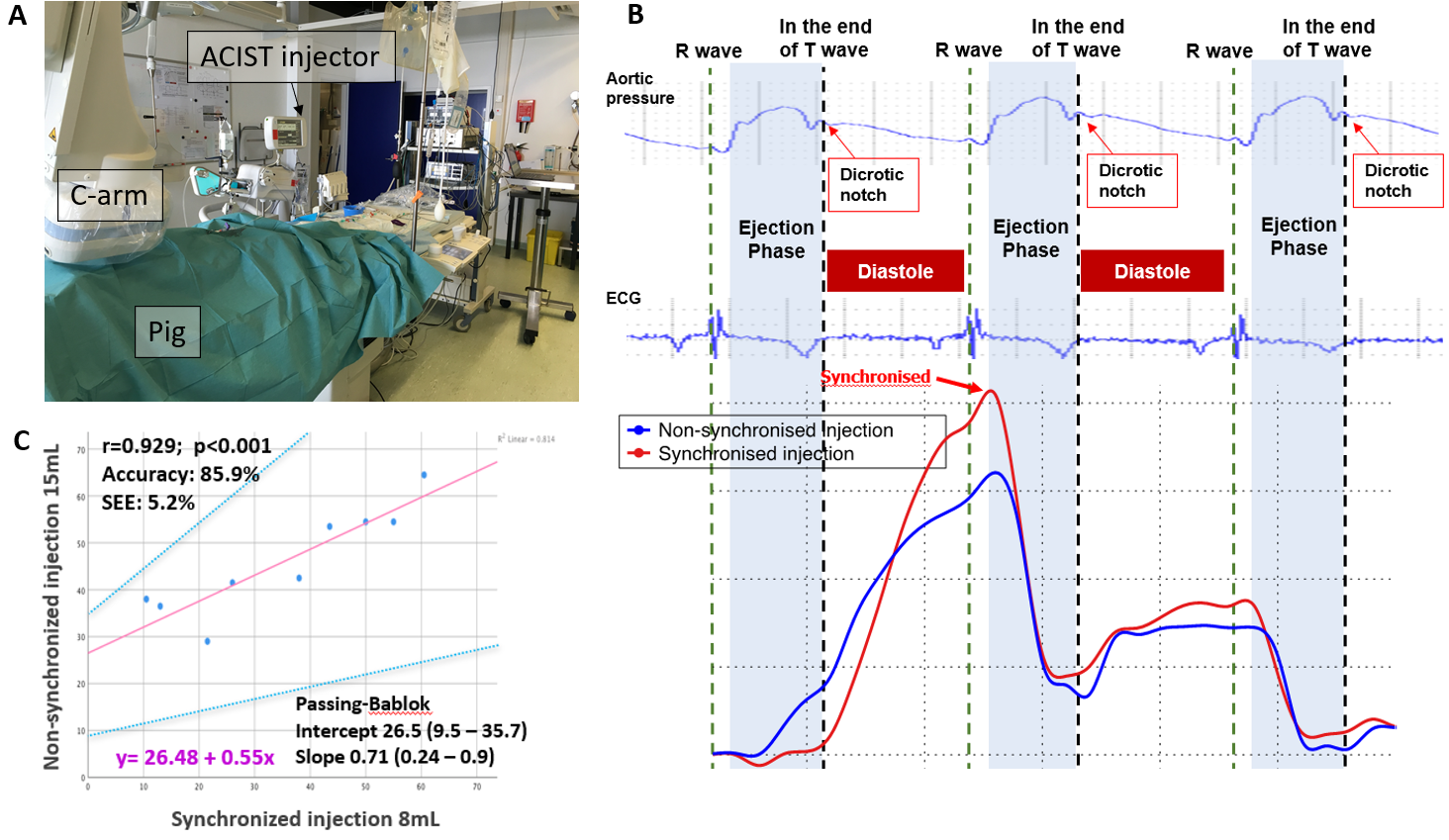


**Supplementary Figure 7**

**
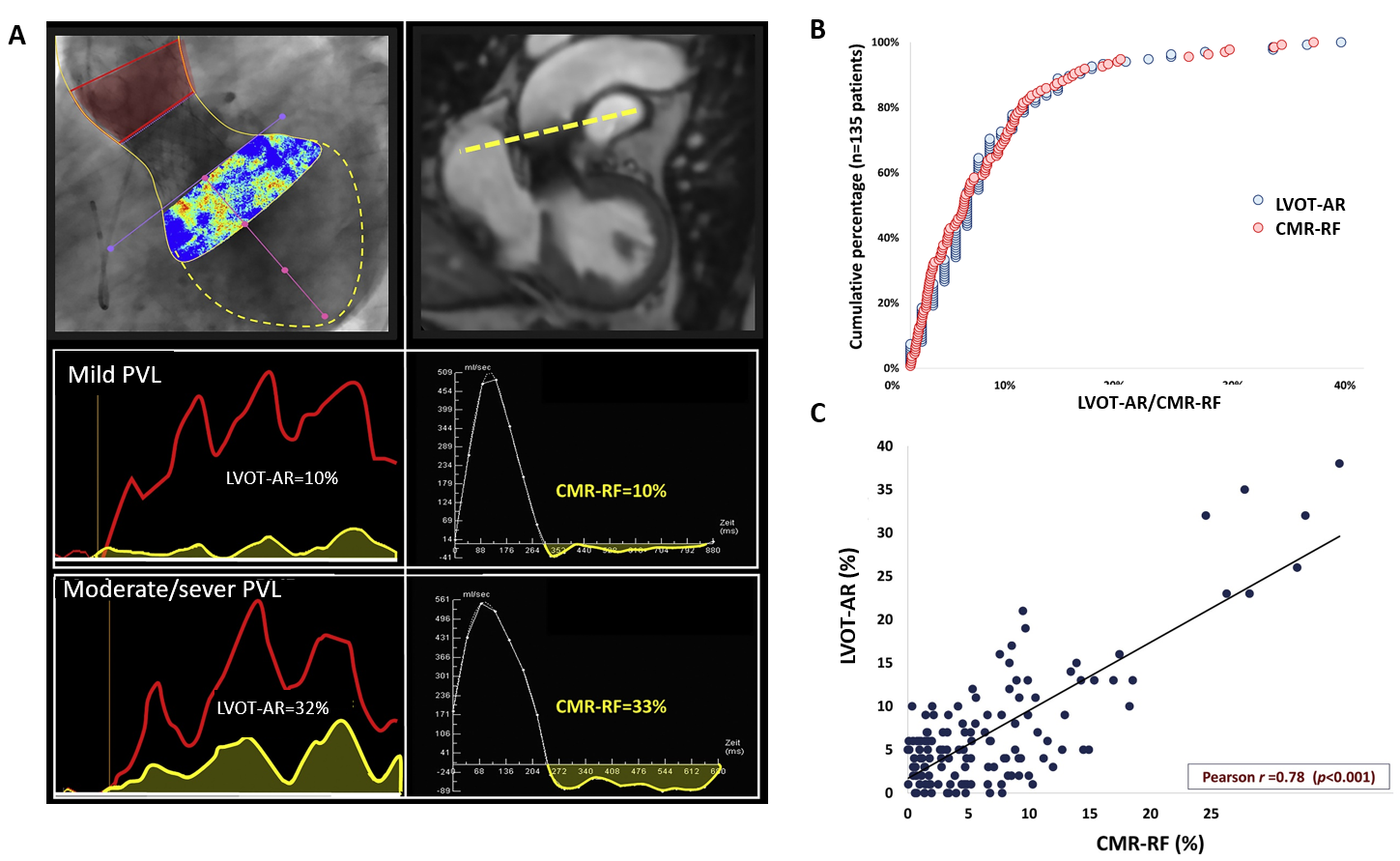
**

**Supplementary Figure 8**


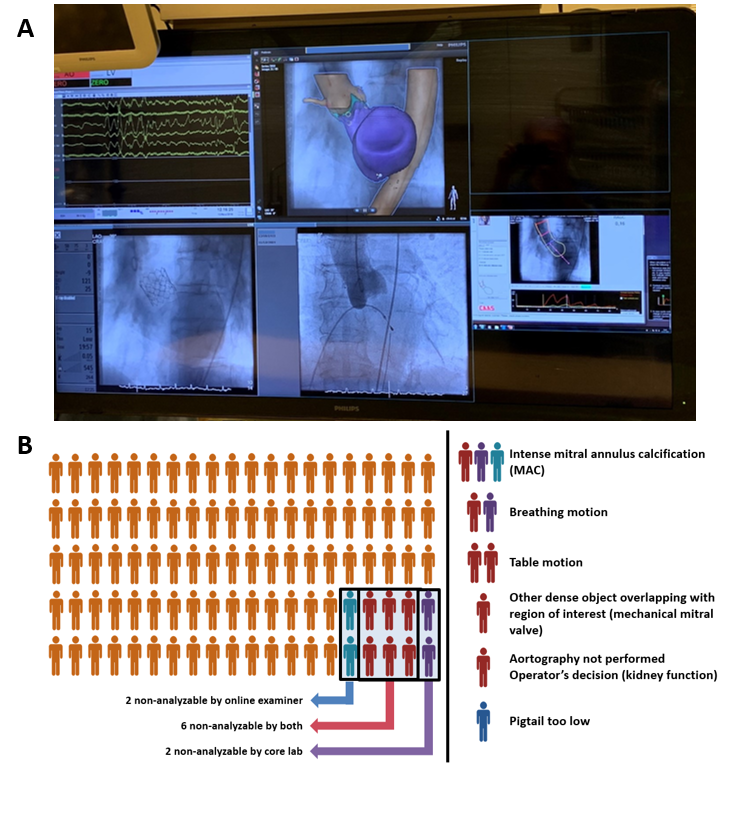


**Supplementary Figure 9**

**
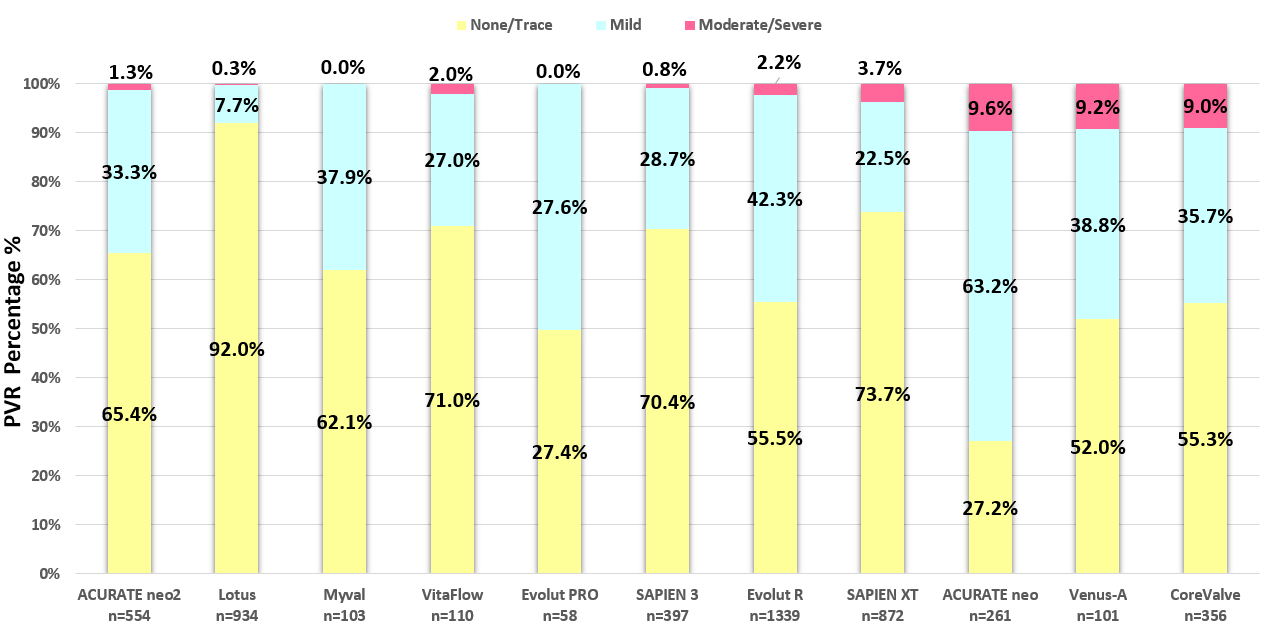
**

**Supplementary Figure 10**


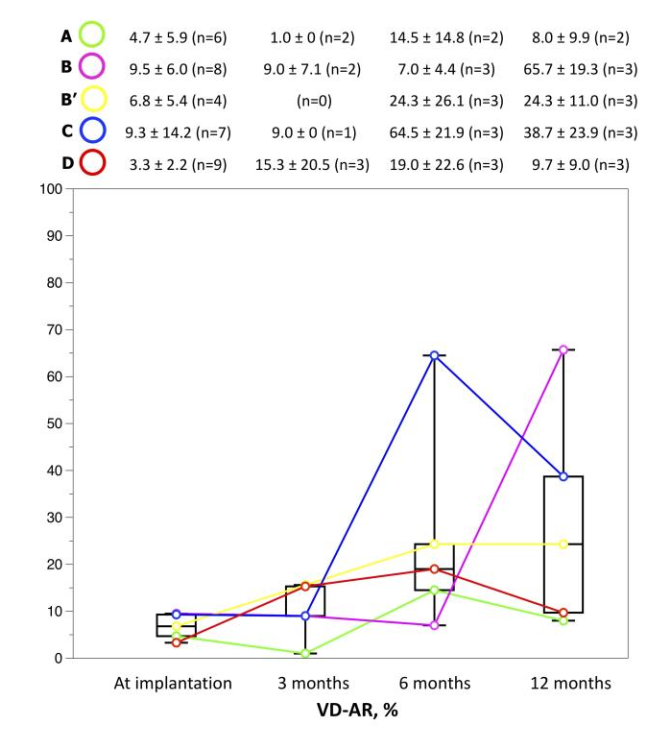


**Supplementary Figure 11.**


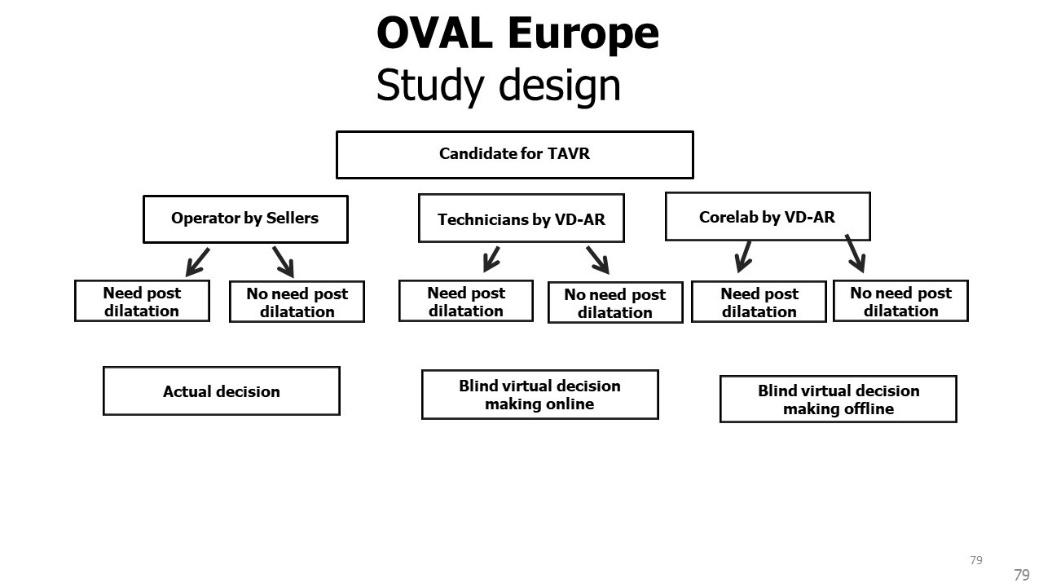


**Supplementary Figure 12.**


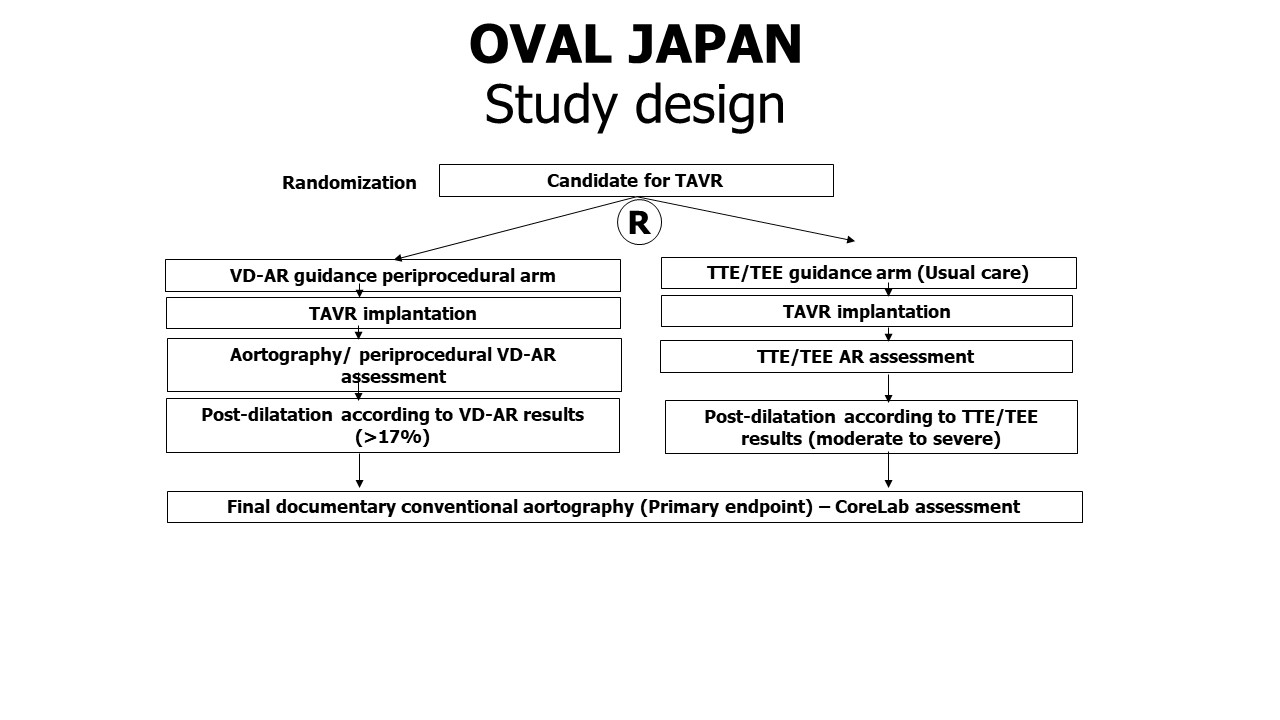


**Supplementary Figure 13**

**
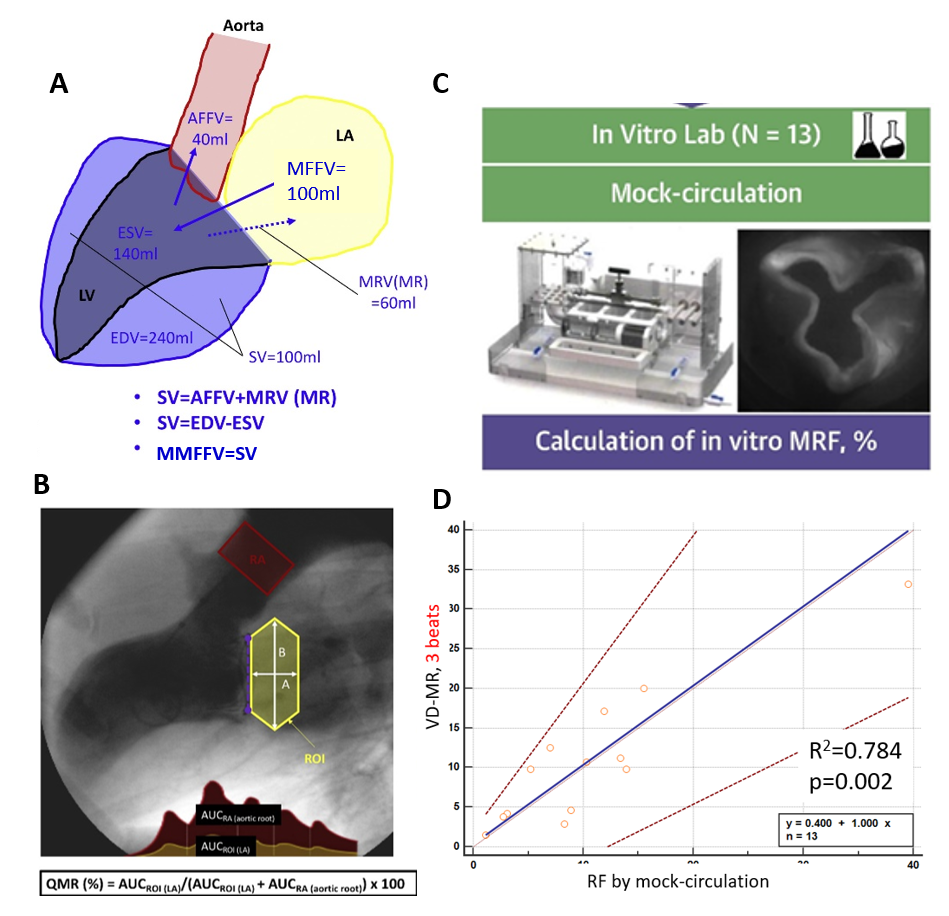
**

**Supplementary Figure 14.**

**
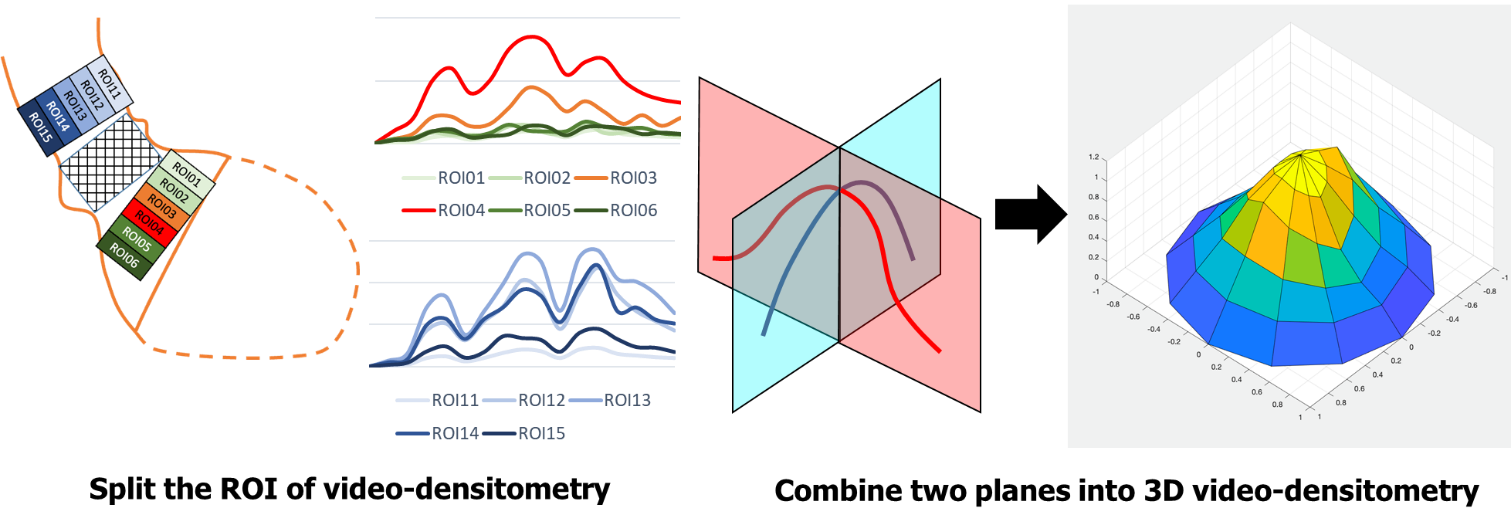
**
